# Leaf cell-specific and single-cell transcriptional profiling reveals a role for the palisade layer in UV light protection

**DOI:** 10.1093/plcell/koac167

**Published:** 2022-06-06

**Authors:** Carl Procko, Travis Lee, Aleca Borsuk, Bastiaan O R Bargmann, Tsegaye Dabi, Joseph R Nery, Mark Estelle, Lisa Baird, Carolyn O’Connor, Craig Brodersen, Joseph R Ecker, Joanne Chory

**Affiliations:** Plant Biology Laboratory, Salk Institute for Biological Studies, La Jolla, California 92037, USA; Plant Biology Laboratory, Salk Institute for Biological Studies, La Jolla, California 92037, USA; Genomic Analysis Laboratory, Salk Institute for Biological Studies, La Jolla, California 92037, USA; Howard Hughes Medical Institute, Salk Institute for Biological Studies, La Jolla, California 92037, USA; School of the Environment, Yale University, New Haven, Connecticut 06511, USA; Biological Sciences, University of California, San Diego, California 92093, USA; Plant Biology Laboratory, Salk Institute for Biological Studies, La Jolla, California 92037, USA; Howard Hughes Medical Institute, Salk Institute for Biological Studies, La Jolla, California 92037, USA; Genomic Analysis Laboratory, Salk Institute for Biological Studies, La Jolla, California 92037, USA; Biological Sciences, University of California, San Diego, California 92093, USA; Department of Biology, University of San Diego, San Diego, California 92110, USA; Flow Cytometry Core Facility, Salk Institute for Biological Studies, La Jolla, California 92037, USA; School of the Environment, Yale University, New Haven, Connecticut 06511, USA; Plant Biology Laboratory, Salk Institute for Biological Studies, La Jolla, California 92037, USA; Genomic Analysis Laboratory, Salk Institute for Biological Studies, La Jolla, California 92037, USA; Howard Hughes Medical Institute, Salk Institute for Biological Studies, La Jolla, California 92037, USA; Plant Biology Laboratory, Salk Institute for Biological Studies, La Jolla, California 92037, USA; Howard Hughes Medical Institute, Salk Institute for Biological Studies, La Jolla, California 92037, USA

## Abstract

Like other complex multicellular organisms, plants are composed of different cell types with specialized shapes and functions. For example, most laminar leaves consist of multiple photosynthetic cell types. These cell types include the palisade mesophyll, which typically forms one or more cell layers on the adaxial side of the leaf. Despite their importance for photosynthesis, we know little about how palisade cells differ at the molecular level from other photosynthetic cell types. To this end, we have used a combination of cell-specific profiling using fluorescence-activated cell sorting and single-cell RNA-sequencing methods to generate a transcriptional blueprint of the palisade mesophyll in *Arabidopsis thaliana* leaves. We find that despite their unique morphology, palisade cells are otherwise transcriptionally similar to other photosynthetic cell types. Nevertheless, we show that some genes in the phenylpropanoid biosynthesis pathway have both palisade-enriched expression and are light-regulated. Phenylpropanoid gene activity in the palisade was required for production of the ultraviolet (UV)-B protectant sinapoylmalate, which may protect the palisade and/or other leaf cells against damaging UV light. These findings improve our understanding of how different photosynthetic cell types in the leaf can function uniquely to optimize leaf performance, despite their transcriptional similarities.

## Teaser

Leaf cell sorting and scRNA-seq approaches are used to transcriptionally profile the palisade mesophyll layer and provide resources for understanding mesophyll biology.

In a Nutshell **Background:** Photosynthesis is arguably the most important biochemical process in plants, and provides humans and other animals with food and oxygen. This process generally occurs in specialized cell types in the leaf. One type of photosynthetic cell, called palisade, forms one or more cell layers at the top of the leaf in many plant species. Despite the importance of these cells, we know surprisingly little at the molecular level as to how palisade differs from other photosynthetic cell types.

**Question:** We wanted to compare the gene expression profile of palisade cells to other photosynthetic cell types to discover their unique roles in leaf biology. We did this by using a combined approach of bulk palisade cell purification and single cell analysis in the model plant *Arabidopsis thaliana*.

**Findings:** Despite their unique morphology and placement in the leaf, gene expression was similar between the palisade and other photosynthetic cell types. Nevertheless, our complementary approaches revealed a role for the palisade in production of an ultraviolet (UV) light-absorbing molecule called sinapoylmalate, which acts as a “sunscreen” to reduce the damaging effects of UV light. This may serve to protect the palisade and/or additional cell types in the leaf. These results provide a better understanding of how different leaf cells function to optimize overall leaf performance.

**Next steps:** In the course of our studies we generated novel genetic tools to probe the function of the palisade and other photosynthetic cells. Future cell-specific manipulations using these tools will further explore how these cells contribute to leaf function across different environments. This will provide insights into why and how leaves adopt different cellular architectures.

## Introduction

For nearly all vascular plants, leaves are the primary sites of light acquisition and photosynthesis. These processes occur primarily in chloroplast-rich mesophyll cells, which make up the bulk of the leaf interior. In laminar leaves, these cells are generally of two types: palisade mesophyll cells, which form one or more layers of columnar cells on the adaxial leaf side, and spongy mesophyll cells, which are arranged below the palisade with a range of irregular to highly ordered morphologies (Haberlandt, 1914; Esau, 1977; Borsuk et al., 2022). The shape and arrangement of these cells are thought to facilitate light capture and CO_2_ movement (Smith et al., 1997; Terashima et al., 2011). Specifically, columnar palisade cells likely conduct excess light deep into the leaf (Vogelmann, 1993) where it is scattered by the spongy mesophyll, acting as a light trap. Additional anatomical properties of leaves, such as the shape of epidermal cells (Vogelmann et al., 1996; Brodersen and Vogelmann, 2007) and placement of stomata mostly on the abaxial surface, further tailor leaf performance to the environment (Smith et al., 1997; Borsuk and Brodersen, 2019; Borsuk et al., 2022).

Of the two mesophyll types, incident sunlight will generally contact the upper palisade cells first. Not surprisingly then, the shape of these cells is particularly affected by light (Hansen, 1917). In high light, additional palisade layers may be present, and the cells adopt a taller, more cylindrical morphology with tighter packing. Experiments using the model plant *Arabidopsis thaliana* (Arabidopsis) suggest these changes are dependent on the blue light sensor phototropin 2 (Kozuka et al., 2011). Other factors regulating leaf adaxial–abaxial asymmetry have similarly been identified from mutant screens in Arabidopsis, and lesions in these genes result in alterations to palisade and/or spongy mesophyll identity (Manuela and Xu, 2020). However, far less is known about the unique molecular signature of mature palisade cells compared with other photosynthetic cell types, and to what extent these cells even differ from each other.

To this end, we sought to transcriptionally profile palisade mesophyll. Reporter lines driving fluorescent proteins in specific cell types have previously been used to sort and profile cells of the root and other organs of Arabidopsis using fluorescence-activated cell (FAC) sorting coupled with microarray or RNA-sequencing (RNA-seq) analysis (Bargmann and Birnbaum, 2010). More recently, single-cell RNA-seq (scRNA-seq) technologies have also been applied to plants (Ryu et al., 2019; Shulse et al., 2019; Liu et al., 2020; Kim et al., 2021; Lopez-Anido et al., 2021), which, in principle, negate the need for specialized reporters and can assess gene expression in all cells of an organ simultaneously. Here, we use both approaches to analyze gene expression in the palisade. Although these cells have unique morphology and appearance, gene expression was strikingly similar to that of other photosynthetic cell types, likely a consequence of their shared primary function. Despite this, we describe novel marker genes for the palisade and characterize a previously unappreciated role for the phenylpropanoid pathway in palisade tissue.

## Results

### FAC sorting of a palisade mesophyll cell population

**Figure 1:**
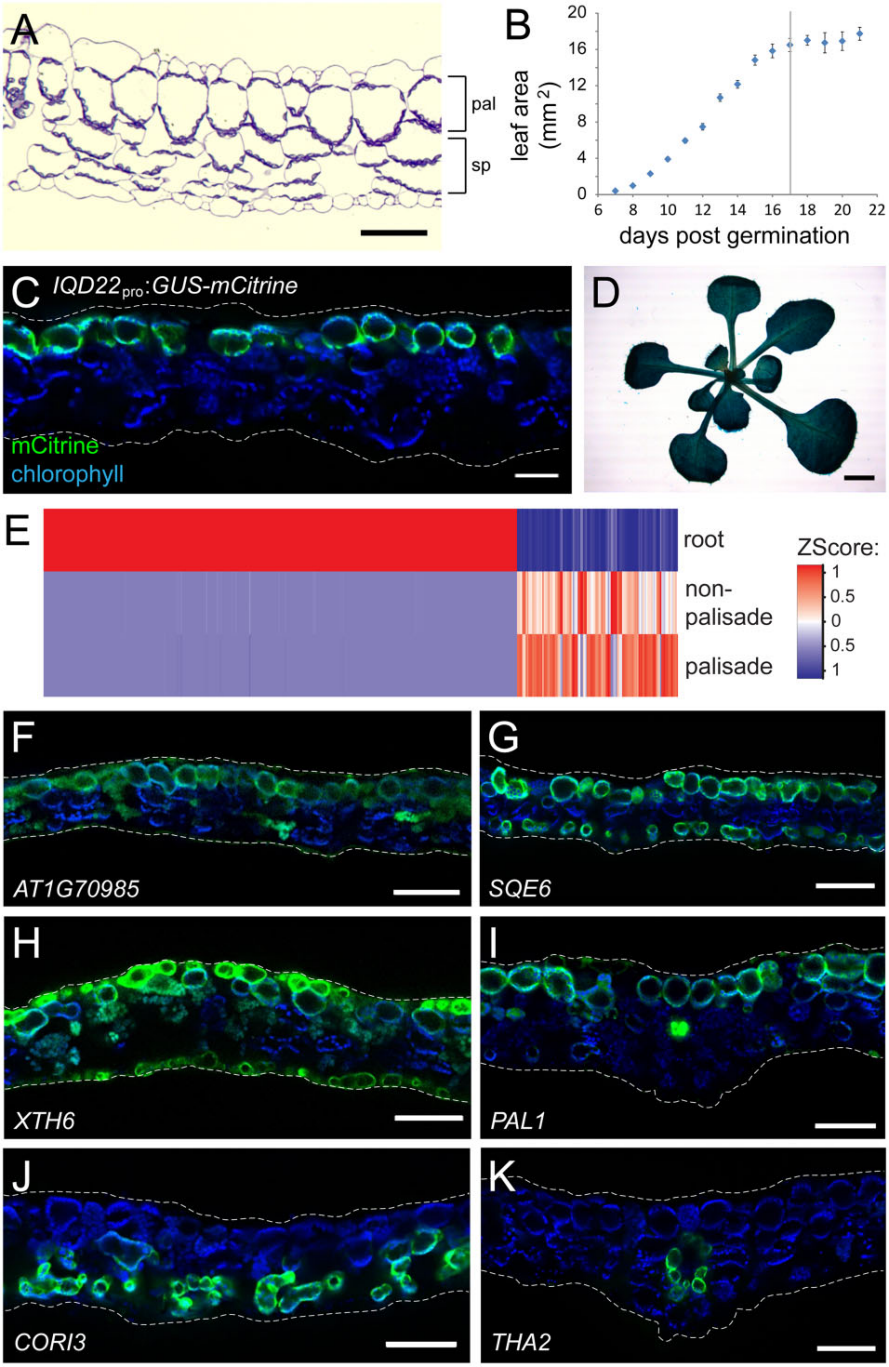
FAC sorting of *IQD22*_pro_:*GUS-mCit* leaf protoplasts can enrich for palisade mesophyll cells. A, Toluidine blue-stained cross-section of a mature Arabidopsis leaf (first true leaf pair), showing a single layer of palisade mesophyll (pal) and spongy mesophyll (sp) below. B, Growth of the first true leaves. In our conditions, the leaves reached their maximum area at 17 days postgermination (vertical line). Mean ± sem. C, Fluorescence image of a leaf cross-section from a 17-day-old *IQD22*_pro_:*GUS-mCit* reporter plant. Chlorophyll autofluorescence is shown in blue and highlights chloroplasts of the mesophyll cells. GUS-mCit protein (green) is expressed in palisade cells. D, Image of the shoot of a GUS-stained *IQD22*_pro_:GUS-mCit plant at day 17. E, Heatmap comparing expression of the top 500 most differentially-regulated genes (columns) identified from pairwise comparisons between total root protoplasts and FAC sorted palisade and nonpalisade photosynthetic cell populations from *IQD22*_pro_:*GUS-mCit* leaves. F–K, Fluorescence images of example transcriptional reporters of genes identified as being upregulated (F–I) or downregulated (J and K) in palisade mesophyll compared with nonpalisade photosynthetic cells by FAC sorting. Leaf cross sections of 17-day-old T1 plants are shown (*n* ≥ 5). Chlorophyll autofluorescence, blue, and gene promoter:*GUS-mCit* reporter, green. Note reporter activity in the palisade cells in (F–K), as well as additional cell types. (I) and (K) are at the location of the midvein; other panels are lateral. Note that when imaging mCit at high exposures in (F) and (H), some autofluorescence is also observed from the cuticle and chlorophyll. Scale bars: (A) and (C), 50 μm; (D), 2 mm; (F–K), 100 μm. In (A), (C), and (F–K), adaxial is up. In (C) and (F–K), dashed white lines show approximate position of cuticle.

To better characterize the palisade layer, we sought to find DNA regulatory sequences in Arabidopsis that drive expression of a fluorescent marker protein specifically in these cells. Arabidopsis leaves show a typical laminar structure, with a single palisade layer directly below the adaxial epidermis and spongy mesophyll below the palisade (Figure 1A). Under our growth conditions, first true leaf expansion was mostly complete by 17 days postgermination (Figure 1B), and, unless otherwise indicated, we performed all subsequent experiments at this developmental stage to assess transcription in the mature leaf.

Work by others previously described β-glucuronidase (GUS) enzyme activity in the palisade layer of the cotyledons in the UCR6 GUS enhancer trap line (Geisler et al., 2002). As such, we sought to better characterize these plants. Using inverse PCR (iPCR), we mapped the *GUS*-coding transposable element insertion site to a region between two genes on chromosome 4. Approximately 4 kb of sequences flanking the insertion site were sufficient to drive expression of the fluorescent reporter protein mCitrine (mCit) in leaf palisade cells, as well as cells in other regions of the plant (Supplemental Figure S1, A and B). We further narrowed down the regulatory sequence to a ∼3.5 kb promoter element upstream of the *IQ-DOMAIN 22* (*IQD22*) gene, and showed that in the leaf this drives accumulation of a GUS-mCit fusion protein in the palisade (*IQD22*_pro_:*GUS-mCit*, Figure 1, C and D and Supplemental Figure S1, C–F).

Using FAC sorting, we generated protoplasts from first true leaves of *IQD22*_pro_:*GUS-mCit* plants, and sorted them based on mCit fluorescence in green wavelengths. In addition, we took advantage of the natural autofluorescent properties of chlorophyll in red wavelengths to further separate chloroplast-containing cells from other, nonphotosynthetic cell types. Using this approach, we purified two cell populations: (1) a chlorophyll-containing population that expressed GUS-mCit, representing the palisade cells and (2) a reference population containing chlorophyll but not displaying GUS-mCit fluorescence (Supplemental Figure S2, A–D). This reference population likely includes spongy mesophyll, bundle sheath cells, and other chlorophyll-containing cells of the vasculature (Haberlandt, 1914; Crookston and Ozbun, 1975; Ding et al., 2015). Here, we assume that this reference population is photosynthetic. RNA-seq suggests that our two populations are transcriptionally very similar: when compared with total root protoplasts, the greatest differences in gene expression are between root cells and photosynthetic cell populations, and not between the photosynthetic cells themselves (Figure 1E and Supplemental Figure S2G). This similarity between photosynthetic cell types is supported by our scRNA-seq analysis (see below). Using these methods, we identified several hundred differentially regulated genes in the palisade cells relative to other photosynthetic cell types, including a known abaxial patterning factor (Supplemental Figure S2, H–J).

To validate our approach, we performed a similar analysis of sorted protoplasts generated from the *GAL4-VP16* enhancer trap line JR11-2, which expresses a modified GFP reporter protein highest in the lower spongy mesophyll (Supplemental Figure S2, E and F; Gardner et al., 2009). FAC-sorted protoplasts from JR11-2 plants divided into a spongy mesophyll-like population (exhibiting both chlorophyll and GFP fluorescence) and a nonspongy, chlorophyll-containing population (exhibiting chlorophyll autofluorescence but lacking GFP, which includes the palisade cells), showed significant overlap with our results from *IQD22*_pro_:*GUS-mCit* plants, thereby validating our methods (Supplemental Figure S2, G–I). However, we were able to detect a larger number of differentially regulated genes using *IQD22*_pro_:*GUS-mCit* generated protoplasts, perhaps due to greater purification of the palisade cells, and thus we confined subsequent analyses to this line.

Finally, to minimize the effect on transcription of the protoplasting process itself, we investigated the effect of adding transcription inhibitors to our protoplasting medium. We found that inhibitors minimized the induction of genes associated with wounding and defense (Supplemental Figure S2, K and L), and thus were added to all subsequent experiments.

Our final analysis found 238 nuclear-encoded genes to be upregulated and 591 downregulated in the palisade population when compared with other photosynthetic cell types (false discovery rate (FDR) < 0.05 and fold-difference > 2; Supplemental Data Sets S1 and S2). To confirm that these genes are palisade enriched, we fused ∼1.5–2 kb promoter elements from a selection of these to a *GUS-mCit* reporter sequence. Transgenic plants carrying these reporters showed diverse expression patterns, some of which had higher expression in the palisade than other photosynthetic cells (8 of 17 reporters tested; Supplemental Figure S3). Examples of these include the promoters for: *AT1G70985*, which drove weak expression mostly in the palisade (Figure 1F); *SQUALENE MONOXYGENASE 6* (*SQE6*), which drove high expression in the palisade and single layer of lowermost spongy mesophyll (Figure 1G); *XYLOGLUCAN ENDOTRANSGLUCOSYLASE/HYDROLASE 6* (*XTH6*), which in addition to the palisade had epidermal expression highest on the adaxial side (Figure 1H); and genes related to phenylpropanoid biosynthesis, including *PHENYLALANINE AMMONIA-LYASE 1* (*PAL1*) (Fraser and Chapple, 2011), which drove highest expression in the vasculature, strong expression in palisade, weak in epidermis, including stomata, and almost none in spongy mesophyll (Figure 1I). By comparison, promoter elements of genes downregulated in the palisade population drove higher expression in other chloroplast-containing cells of the leaf. Examples include the promoters for: *CORONATINE INDUCED 3* (*CORI3*), which drove expression in the lower spongy mesophyll and bundle sheath (Figure 1J); *THREONINE ALDOLASE 2* (*THA2*) in bundle sheath (Figure 1K); and other gene promoter elements that drove expression in spongy mesophyll, bundle sheath and/or chloroplast containing cells of the vasculature (Supplemental Figure S4).

Some of our reporters for genes predicted to be palisade-enriched failed to express in these cells (Supplemental Figure S3). This may reflect that we have inadequate promoter/regulatory sequences for these particular genes. For example, we were unable to detect GUS-mCit activity from a plant carrying an *LHCB4.3*_pro_:*GUS-mCit* reporter; however, a previous study suggested that this gene is expressed on the adaxial leaf side (Sawchuk et al., 2008). Similarly, while we were able to identify expression of the phenylpropanoid-related gene *FERULIC ACID 5-HYDROXYLASE 1* (*FAH1*) in FAC sorted palisade cells (see below), regulatory elements required for *FAH1* gene function in the leaf lie not within the promoter sequence but rather in a ∼12.5 kb region downstream of the stop codon (Ruegger et al., 1999). Alternatively, our FAC sorted palisade population may contain contaminating cells. Nevertheless, our results suggest that our methods have successfully purified the palisade cells, at least partially.

### scRNA-seq of the mature Arabidopsis leaf

Recently, scRNA-seq methods have been developed as an alternative approach to query specific cell populations. Previously reported scRNA-seq analyses of Arabidopsis cotyledons, immature developing leaves, and leaves of mature 6-week-old plants did not distinctly separate the palisade and spongy mesophyll (Liu et al., 2020; Kim et al., 2021; Lopez-Anido et al., 2021), possibly as a result of the high transcriptional similarity we have observed. As such, to complement our FAC sorting experiments, we performed scRNA-seq using the 10× Genomics platform on protoplasts generated from the first true leaves of 17-day-old plants carrying the *IQD22*_pro_:*GUS-mCit* reporter, so as to improve our ability to identify the palisade population.

**Figure 2:**
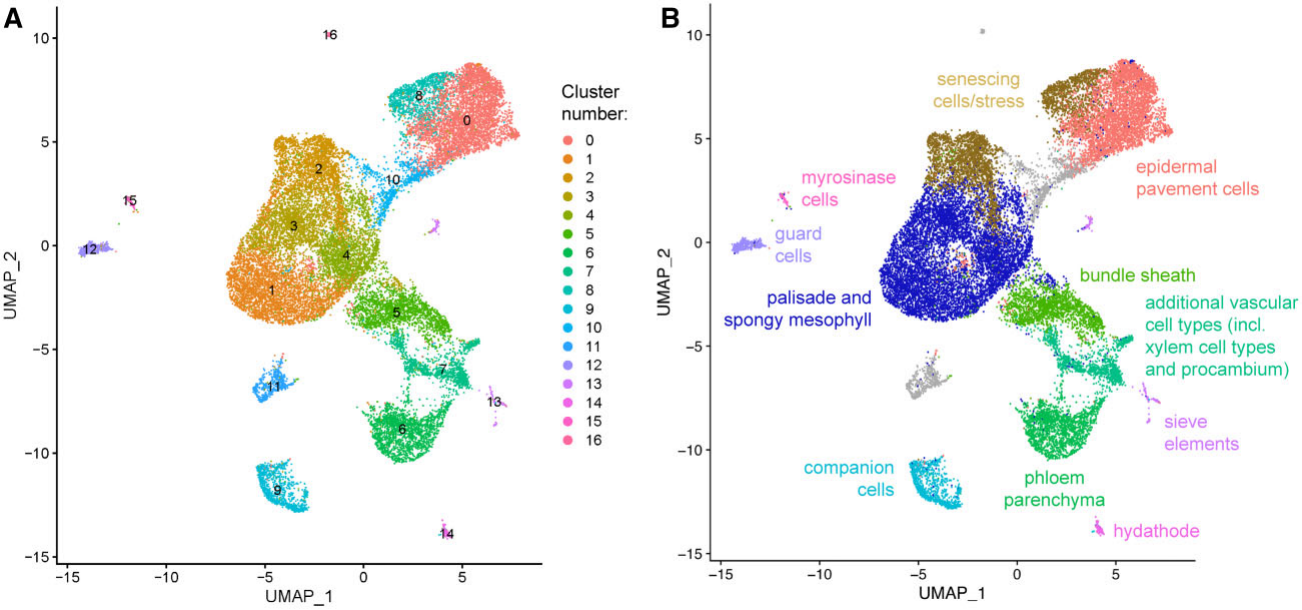
A scRNA-seq atlas of mature Arabidopsis leaf protoplasts. A, Unbiased clustering of RNA-sequenced leaf protoplasts from *IQD22*_pro_:*GUS-mCit* plants. 17 unique cell clusters were assigned based on transcriptional differences (clusters 0–16). B, Cell identities of UMAP-based clusters determined by marker gene analysis and validation.

**Figure 3:**
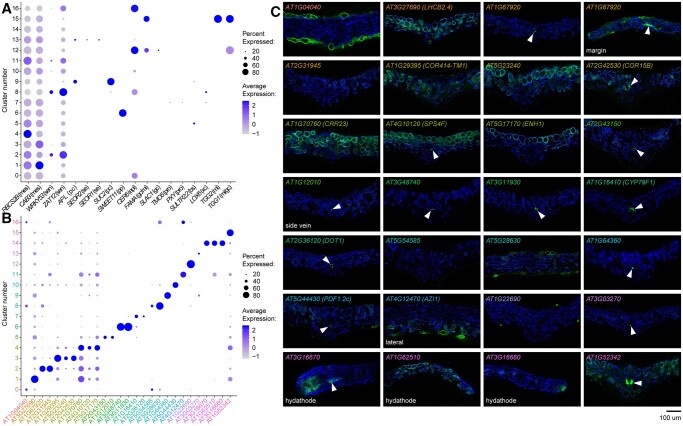
Gene marker analysis of scRNA-seq cell clusters. A, Dot plot showing expression of known tissue-specific marker genes in the scRNA-seq leaf cell clusters (see Figure 2A). Dot color intensity represents average expression of the gene in a given cluster, and dot size the percentage of cells in the cluster expressing that particular marker. Markers are expressed in mesophyll (mes), senescing cells (sen), phloem companion (pc), sieve elements (se), phloem parenchyma (pp), epidermis (epi), GCs (gc), myrosinase idioblasts (mi), procambium (pro), bundle sheath and veins (bs), and xylem contact cells (xc). B, Dot plot showing expression of de novo-identified cluster marker genes. Gene name color is the same as the cell cluster in Figure 2A for which the marker gene was identified. C, Fluorescence images of transcriptional reporters for genes shown in (B). Cross sections of first true leaves of T1 transgenic plants are shown (representative from *n* ≥ 4 T1 plants). Chlorophyll autofluorescence, blue, and gene promoter:*GUS-mCit* reporter activity, green. Adaxial is up. All images are at the midvein except where otherwise indicated (margin, side vein, lateral or hydathode). Arrowheads mark vascular and/or bundle sheath expression in some lines.

Following doublet removal (DePasquale et al., 2019) and filtering, our scRNA-seq dataset included 23,729 cells, with 3,639 median unique transcripts per cell and 1,637 median genes detected per cell. While these median counts are generally lower or comparable to other similar studies from plants, the number of cells in our dataset are several fold higher (Supplemental Table S1). Using Uniform Manifold Approximation and Projection (UMAP) for dimensionality reduction, we identified 17 cell clusters corresponding to unique cell types and/or transcriptional states (Figure 2A). More stringent filtering of our data set to remove cells with > 10,000 unique molecular identifiers (UMIs) per cell generated similar results, suggesting that the inclusion of some cells with high RNA content is not affecting our overall findings (Supplemental Figure S5). To assign biological identity to these (Figure 2B) we used two approaches. First, we used a biased approach, by mapping known cell type marker genes to the UMAP plot (Figure 3A). Second, we used an unbiased method. For this, we fused ∼2 kb of promoter elements for one or more genes identified through our analyses as being differentially upregulated in a given cluster to the *GUS-mCit* coding sequence, and imaged leaves of T1 transformed plants in cross-section (Supplemental Data Sets S3–S19, Figure 3, B and C, and Supplemental Figure S6).

Using these combined approaches, we associated several clusters with cell types of veins. Known marker genes *SWEET11* (Chen et al., 2011), *ALTERED PHLOEM DEVELOPMENT* (*APL*) and *SUCROSE TRANSPORTER 2* (*SUC2*) (Truernit and Sauer, 1995; Bonke et al., 2003), *SIEVE ELEMENT OCCLUSION-RELATED 1* and *2* (*SEOR1* and *SEOR2*) (Rüping et al., 2010; Anstead et al., 2012), and *THIOGLUCOSIDE GLUCOHYDROLASE 2* (*TGG2*) (Barth and Jander, 2006), expressed in phloem parenchyma, phloem companion cells, sieve elements, and myrosinase idioblasts, respectively, mapped to cluster numbers 6, 9, 13, and 15 (Figure 3A). Procambium markers *TARGET OF MONOPTEROS 5* (*TMO5*) and *PHLOEM INTERCALATED WITH XYLEM* (*PXY*) (Fisher and Turner, 2007; De Rybel et al., 2013), and the xylem contact cell-expressed gene *LIPOXYGENASE 6* (*LOX6*) (Chauvin et al., 2012; Nguyen et al., 2018), mapped to distinct regions of cluster 7, suggesting this cluster represents a mixed population of vascular cell types (Figure 3A). Consistent with these findings, promoter elements from mostly uncharacterized genes associated with clusters 6, 7, 9, 13, and 15 (*AT3G48740*, *AT3G11930*, *AT1G16410*/*CYTOCHROME P450 79F1* (*CYP79F1*; see also Tantikanjana, 2001), *AT2G36120*/*DEFECTIVELY ORGANIZED TRIBUTARIES 1* (*DOT1*), *AT1G64360*, *AT3G03270*, and *AT1G52342*; Figure 3B) all drove *GUS-mCit* reporter activity in cells associated with minor and/or major veins, and, in the case of *CYP79F1*, weakly in the bundle sheath (Figure 3C and Supplemental Figure S6). Gene ontology (GO) term enrichment analysis (Supplemental Data Sets S20–S36) suggests that these cell clusters express genes involved with glucosinolate production, water transport and phloem transport, among other processes. Cluster 5, which shared similarities in gene expression with cell types of the vein, expressed the bundle sheath and vein marker *SULPHATE TRANSPORTER 2;2* (*SULTR2;2*) (Takahashi et al., 2000; Kirschner et al., 2018), as well as genes related to glucosinolate production, starch biosynthesis and photosynthesis (Figure 3A and Supplemental Data Set S25). Similarly, reporters for novel cluster 5 marker genes *AT2G43150* and *AT1G12010* drove expression in bundle sheath and/or cells of the vein (Figure 3, B and C).

To identify guard cells (GCs), we looked for the overlap in expression between the cuticle-formation gene *ECERIFERUM 6* (*CER6*) (Hooker et al., 2002), the GC-specific gene *SLOW ANION CHANNEL-ASSOCIATED 1* (*SLAC1*) (Vahisalu et al., 2008), and the GC and myrosinase idioblast markers *FAMA* and *THIOGLUCOSIDE GLUCOHYDROLASE 1* (*TGG1*) (Barth and Jander, 2006; Shirakawa et al., 2014; Figure 3A). This identified cluster 12 as GC-specific. As validation, a promoter element for a de novo identified cluster 12 marker, *AT1G22690*, drove reporter expression specifically in stomata (Figure 3, B and C).

To identify clusters 11, 14, and 16, we tested reporter activity of various marker genes identified through our bioinformatic approaches. For cluster 14, promoter elements from markers *AT3G16670*, *AT1G62510*, and *AT3G16660* all drove expression either specifically in the hydathode, or the hydathode and other cell types (Figure 3, B and C). This cluster was enriched for genes related to immune responses among other biological processes, including auxin biosynthesis, known to occur in hydathodes (Biedroń and Banasiak, 2018; Supplemental Data Set S34). In contrast, the marker gene *AT4G12470*/*AZELAIC ACID INDUCED 1* (*AZI1*), involved in systemic immunity (Cecchini et al., 2015), showed enriched expression in clusters 11 and 16 (Figure 3B). Both these clusters express genes related to oxidative stress and stress hormones jasmonic acid and salicylic acid (Supplemental Data Sets S31 and S36). Interestingly, the promoter element of *AZI1* drove reporter activity in a subset of abaxial epidermal cells and mesophyll, and, as such, we were unable to assign a specific cell type or state to these two clusters (Figure 3C). Future study of these populations may resolve their identity, and what specific roles they play in leaf function and defense.

**Figure 4:**
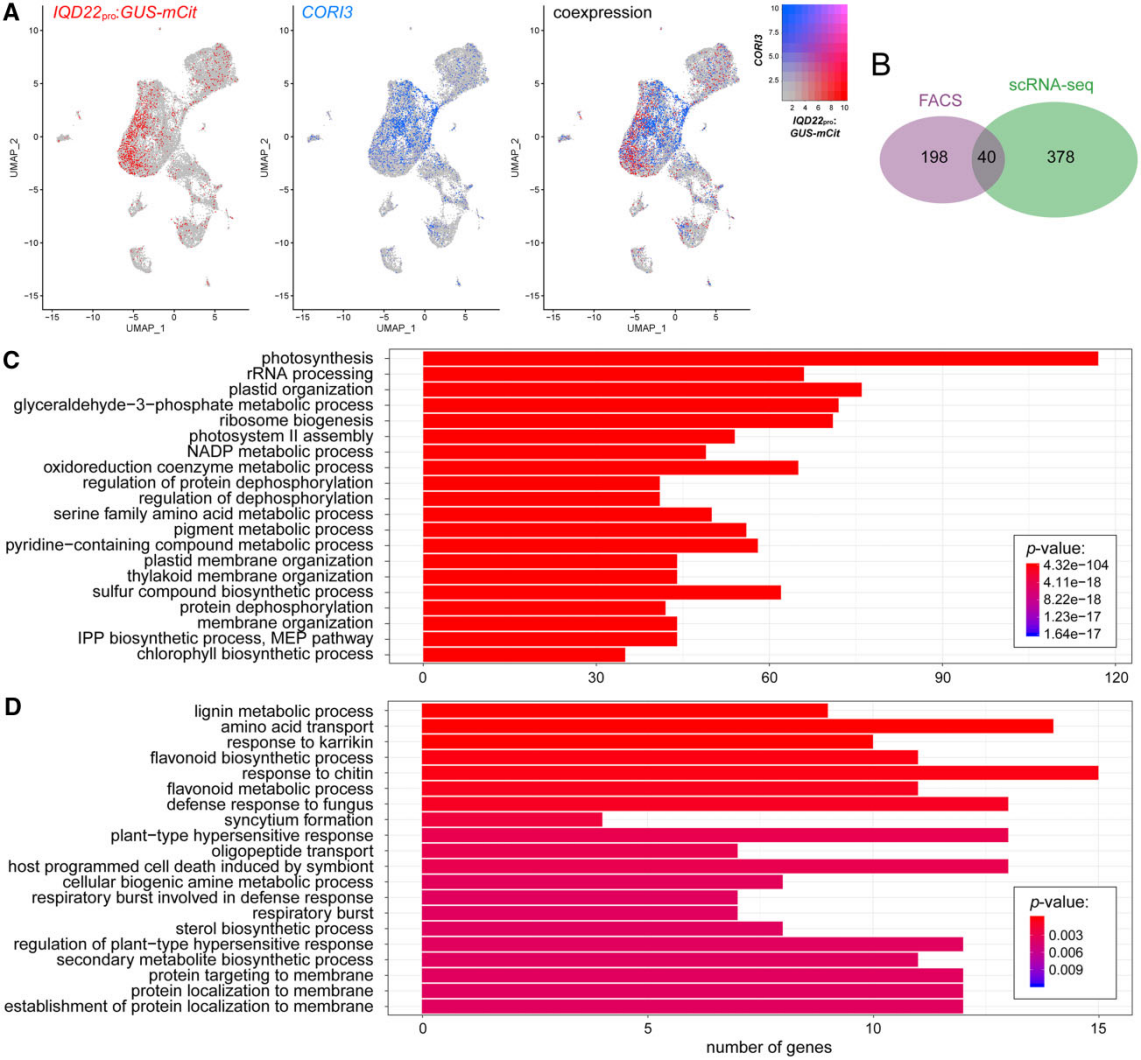
Identification of palisade-expressed genes by scRNA-seq. A, Location of *GUS-mCit* and *CORI3* expressing cells in our scRNA-seq UMAP plot of protoplasts from the mature leaf of *IQD22*_pro_:*GUS-mCit* transgenic plants. Note little overlap of cells expressing these two transcripts. B, Overlap of nuclear-encoded genes identified to be upregulated in the palisade compared with reference photosynthetic cell populations as determined by FAC sorting, and *GUS-mCit* expressing cells (reads > 1) compared with *CORI3* expressing cells (reads > 2) by scRNA-seq methods. *P* < 0.005, hypergeometric test. C and D, GO term enrichment analysis of genes upregulated in the palisade as identified using scRNA-seq (C) or FAC sorting (D). The top 20 terms by adjusted *P*-value are shown.

The two largest groups of cells, representing clusters 0 and 8, and clusters 1–4 (Figure 2A), expressed epidermal pavement and mesophyll/photosynthesis-related genes, respectively. In clusters 0 and 8, this included the cuticle formation gene *CER6*, as well as the newly identified epidermal markers *AT1G04040* and *AT5G28630* (Figure 3, A–C; a reporter for an additional cluster 8 marker, *AT5G54585*, had little expression in the first true leaves). In contrast, photosynthesis-related marker genes *CHLOROPHYLL A/B BINDING PROTEIN 3* (*CAB3*) and *RUBISCO SMALL SUBUNIT 2B* (*RBCS2B*), which are expressed in various regions of the mature leaf (Susek et al., 1993; Sawchuk et al., 2008), had broad expression domains with strongest expression in clusters 1 and 4, respectively (Figure 3A). Interestingly, clusters 2 and 8 shared expression of stress- and senescence-related genes such as *WRKY53* and *ZAT12* (Davletova et al., 2005; Bresson et al., 2018; Zentgraf and Doll, 2019; Figure 3A and Supplemental Data Sets S22 and S28). This may indicate that senescence promotes a transcriptional convergence of epidermal pavement and mesophyll cells irrespective of cell type. A reporter for a cluster 2 marker gene, *AT1G67920*, drove expression in vascular and epidermal cells (Figure 3, B and C), and we hypothesize that this cluster includes mixed cell types undergoing senescence; however, another cluster 2 promoter, *AT2G31945*, failed to drive expression in the leaf blade, perhaps due to low activity or incomplete regulatory sequences. Similarly, abaxial-positioning in the leaf may influence the transcriptome of some epidermal and mesophyll cells into cluster 10, which expresses the spongy mesophyll marker *CORI3*, described above, and a newly identified marker gene for regions of the abaxial epidermis, *AT5G44430*/*PLANT DEFENSIN 1.2c* (*PDF1.2c*) (Figures 3, B and C and 4A).

In contrast, other markers we tested for clusters 1, 3, and 4 were unable to resolve if one or more of these clusters corresponded to a specific mesophyll cell type or not. For example, promoter elements for cluster 4 markers *AT4G10120*/*SUCROSE PHOSPHATE SYNTHASE 4F* (*SPS4F*) and *AT5G17170*/*ENHANCER OF SOS3-1* (*ENH1*) drove expression predominantly in the palisade, as did cluster 3 marker *AT5G23240* (Figure 3, B and C), while *IQD22*_pro_: *GUS-mCit* was detected predominantly in cluster 1 (Figure 4A). Other cluster 1, 3, and 4 markers *AT3G27690*/*LHCB2.4*, *AT1G29395*/*COLD REGULATED 414 THYLAKOID MEMBRANE 1* (*COR414-TM1*) and *AT1G70760*/*CHLORORESPIRATORY REDUCTION 23* (*CRR23*), respectively, drove expression more broadly in the palisade and spongy mesophyll, while the promoter element from another cluster 3 marker, *AT2G42530*/*COLD REGULATED 15B* (*COR15B*), drove expression mostly in bundle sheath. As such, we broadly associate these clusters with mesophyll cell types and possibly some bundle sheath cells. Our and others’ (Liu et al., 2020; Kim et al., 2021; Lopez-Anido et al., 2021) inability to cleanly resolve the mesophyll populations by scRNA-seq, in addition to their close proximity on our UMAP plot, further supports the conclusion that mesophyll cell types are transcriptionally similar to one another, despite their morphological differences.

Interestingly, subclustering of related major clusters may reveal additional cell-types and cell-states. For example, subclustering of epidermal pavement cell clusters 0 and 8 revealed that subpopulations of epidermal cells may be present therein (Supplemental Figure S7). Reporter analysis of marker genes *AT2G39310* and *AT3G09260*/*LONG ER BODY* (*LEB*) suggests that one of these subclusters includes abaxial epidermal cells near the margins and proximal midvein.

### Phenylpropanoid-related genes are expressed in the palisade

The inability to discretely resolve spongy and palisade mesophyll populations in our scRNA-seq study may be due to (1) high levels of similarity between the two cell types (see above); (2) gene expression within the mesophyll being influenced by additional positional information on the proximal–distal and/or medio–lateral axes, or proximity to other leaf structures such as veins; or (3) complexity in cell states; for example, mesophyll cells undergoing various levels of senescence. Alternatively, it may (4) reflect limitations with our reporter-based verification approach. To circumvent these issues, we instead took advantage of the fact that our sequenced leaf protoplasts carried the *IQD22*_pro_:*GUS-mCit* transgene, which should uniquely label the palisade cells. As a reference population, we compared these cells to those with high expression of the abaxial spongy mesophyll marker we identified above, *CORI3* (Figure 4A). Comparing these populations, our scRNA-seq results identified 418 nuclear-encoded genes as being differentially upregulated in *IQD22*_pro_:*GUS-mCit* expressing cells (adjusted *P*-value < 0.05) (Supplemental Data Set S37). Of these, 40 were also identified as upregulated in the palisade in our FAC-sorting experiments, including *IQD22*, with a statistically significant overlap (hypergeometric test, *P* < 0.005; Figure 4B). In addition to *IQD22*, for eight of these genes (*AT4G19200*, *AT1G11210*, *ANTHOCYANINLESS 2* (*ANL2*), *HYDROXYCINNAMOYL-COA SHIKIMATE/QUINATE HYDROXYCINNAMOYL TRANSFERASE* (*HCT*), *AT1G22500*, *HAT3*, *AT1G70985*, and *LHCB4.3*) we had already generated transcriptional reporters (Supplemental Figure S3). The lack of further overlap between the two approaches might reflect differences in the reference cell populations chosen, among other possibilities (see “Discussion”). These results demonstrate that the two approaches detect many of the same transcripts, although the presence of uniquely identified transcripts with each method suggests the two techniques are complementary and might uncover unique information.

GO term enrichment analysis of genes with high expression in the palisade relative to *CORI3* transcript-expressing cells identified using scRNA-seq suggested that many of the differentially regulated genes were related to photosynthesis and chloroplast function (Figure 4C). This included many chlorophyll-a/b binding proteins (Supplemental Data Set S37), which have previously been detected at high levels in the palisade of soybean (*Glycine max*) cotyledons (Chang and Walling, 1992). These results suggest that the palisade may have unique photochemistry. Interestingly, a similar analysis of palisade upregulated genes identified from FAC sorting also suggested high activity of genes associated with lignin and flavonoid metabolism (Figure 4D). Lignin and flavonoid production both originate from precursor molecules synthesized in the phenylpropanoid biosynthetic pathway (Zhang et al., 2021). While these GO terms were not enriched in our list of palisade upregulated genes identified from scRNA-seq, several of these same genes were also found to be upregulated; for example, *CINNAMYL-ALCOHOL DEHYDROGENASE 7* (*CAD7*), *O-METHYLTRANSFERASE 1* (*OMT1*), and *HCT* (Supplemental Data Sets S1 and S37; Fraser and Chapple, 2011). As such, both data sets support high activity of the phenylpropanoid pathway within the palisade layer compared with other photosynthetic cell types.

The inability of scRNA-seq methods to detect as many phenylpropanoid-related transcripts in the palisade may be due to limitations with this method in detecting low abundance transcripts (Saliba et al., 2014). For example, while both scRNA-seq and FAC sorting methods were able to detect our palisade marker *IQD22* and the phenylpropanoid-related transcript *HCT*, only FAC sorting was able to identify enriched palisade expression of another phenylpropanoid gene, *PAL1*, even though we observed *PAL1* reporter expression within this tissue (Figure 1I and Supplemental Figure S8). In the future, deeper coverage may or may not resolve this issue. Because of these limitations, we chose to use FAC sorting methods for subsequent transcriptional profiling experiments.

### Phenylpropanoid genes are induced by light in the palisade

**Figure 5:**
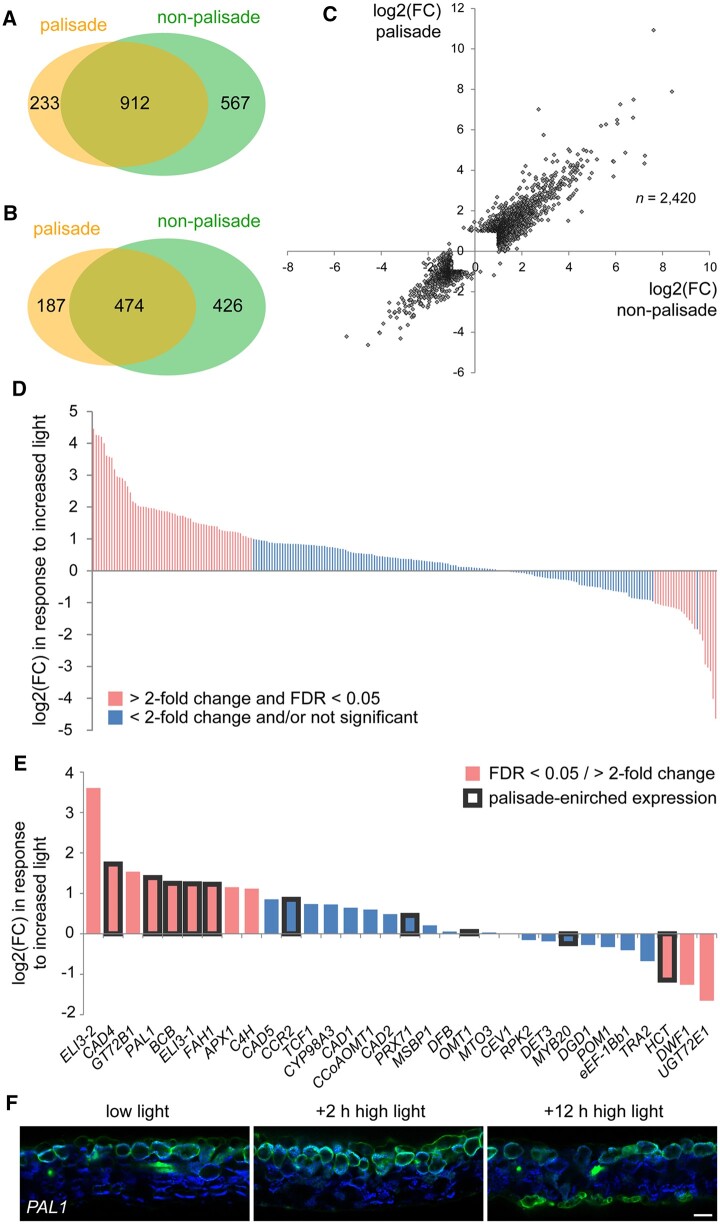
Effect of high light on gene expression in the palisade mesophyll. A and B, A comparison of the genes induced (A) and repressed (B) by high light in FAC sorted palisade cells compared with the photosynthetic reference cell population (FDR < 0.05 and fold change (FC) > 2). *IQD22*_pro_:*GUS-mCit* plants were grown for 17 days at 50 μmol m^−2^ s^−1^. High light treated plants were then moved to 300 μmol m^−2^ s^−1^ for 2 h prior to protoplasting. The two cell populations have a high degree of overlap in the genes that are regulated (*P* < 0.005, hypergeometric test). C, A comparison of the FC in gene expression in response to high light for all genes identified as differentially regulated by light in either the palisade or reference cell population. Note that most genes are regulated to a similar degree in the two populations. D, FC in expression when responding to the high light stimulus for genes identified as upregulated in the palisade mesophyll by FAC sorting (bars on *x*-axis). Genes significantly regulated by light (FDR < 0.05) with FC > 2 are indicated. E, The high light response of genes associated with GO term “lignin biosynthetic process” (GO:0009809) detected in our RNA-seq experiment in the FAC-sorted palisade mesophyll cells. Genes also identified as upregulated in the palisade are boxed in black. F, Representative fluorescence images of first true leaf cross-sections of T1 plants carrying a *PAL1*_pro_:*GUS-mCit* reporter (green). Plants were grown at 50 μmol m^−2^ s^−1^ (low light) for 17 days then shifted to 300 μmol m^−2^ s^−1^ (high light) for 2 or 12 h prior to imaging (*n* = 6 T1 plants each condition). Images were taken at the same exposure. Note a shift in reporter expression to the abaxial epidermis after 12-h high light. Chlorophyll autofluorescence, blue. Scale bar: 50 μm. Adaxial is up.

High light is known to regulate both gene expression and palisade cell morphology (Hansen, 1917; Huang et al., 2019). Therefore, to test the effect of light specifically on the palisade layer, we performed RNA-seq of FAC sorted protoplasts from *IQD22*_pro_:*GUS-mCit* plants treated for 2 h with increased light fluence rate. This treatment did not alter the fluorescent reporter used for sorting (Supplemental Figure S9). We observed that high light had a profound effect on both palisade mesophyll and the reference cells, with over a thousand genes being upregulated or downregulated in both populations (Supplemental Data Sets S38–S41). However, like observations we made above, we saw few differences between them, further reinforcing that different mesophyll types have very similar transcriptional programs and also respond similarly to the light environment (Figure 5, A–C). Interestingly, we also observed that many palisade-enriched genes identified via FAC sorting were regulated by high light, including phenylpropanoid pathway genes associated with GO terms related to lignin and flavonoid metabolism (Figure 5, D and E and Supplemental Figure S9E). This is consistent with the observation that ultraviolet (UV) light induces phenylpropanoid pathway activity (Dixon and Paiva, 1995; Demkura and Ballaré, 2012). The genes induced by high light included *PAL1*, which was predicted to be upregulated ∼2.6-fold in the palisade cells following the 2 h high light treatment. While we were unable to verify this increase in expression with transgenic *PAL1*_pro_:*GUS-mCit* reporter plants due to the qualitative nature of our imaging assay and variation among T1 plants, we did observe a shift in expression from the palisade to the abaxial epidermis following a longer 12-h high light treatment (Figure 5F). This was in addition to the vascular and adaxial epidermal expression we reported above. This greater expression in the abaxial epidermis under prolonged high light may correlate with flavonoid pigment production (Lois, 1994; Hughes and Smith, 2007; Huang et al., 2010), and further supports the conclusion that phenylpropanoid genes are dynamically regulated by light. Because of this observation, as well as the observation that a number of phenylpropanoid genes had palisade-enriched expression under our low light conditions, we further investigated the role of this pathway in the palisade.

### Phenylpropanoid gene expression in the palisade is required for sinapoylmalate production

To better understand the role of phenylpropanoid biosynthesis in palisade tissue, we took advantage of mutant plants carrying a lesion in the *FAH1* gene, which encodes ferulate-5-hydroxylase (Chapple et al., 1992; Meyer et al., 1996). Mutations in *FAH1* result in a reduction in two end products of the phenylpropanoid pathway: (1) sinapic acid-derived syringyl lignin (S lignin), and (2) sinapate esters, including sinapoylmalate in leaves (Chapple et al., 1992). Importantly, sinapoylmalate functions as a UV-B light absorbant and protectant, and *fah1* mutants are sensitive to increased UV (Landry et al., 1995). When visualized under UV light, the leaves of *fah1* mutants fluoresce red due to increased absorbance of UV light by chlorophyll pigments (Chapple et al., 1992).

**Figure 6:**
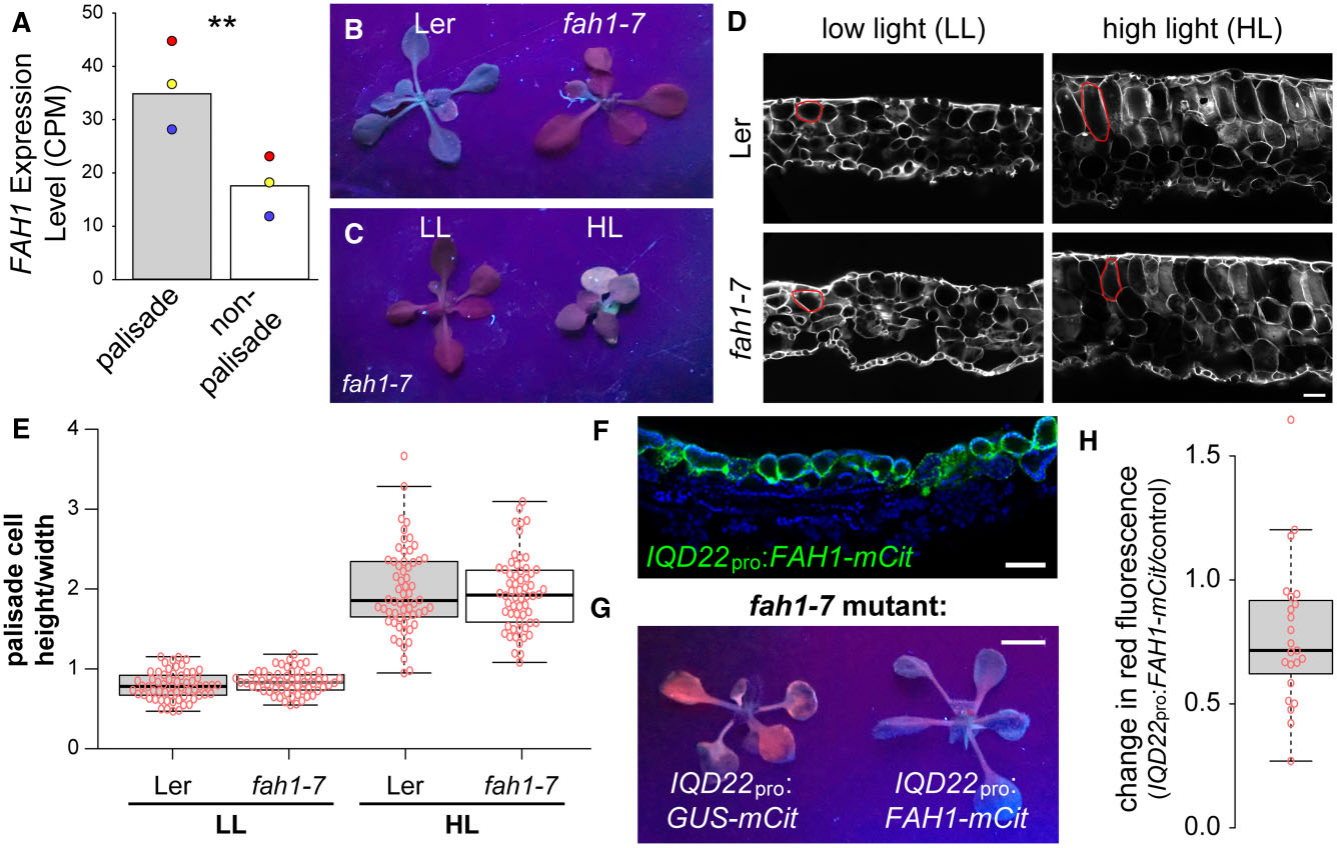
*FAH1* expression in the palisade is required for sinapoylmalate accumulation. A, *FAH1* gene expression by counts per million (CPM) in FAC sorted palisade mesophyll and photosynthetic reference cell populations. Colored dots indicate values from the three paired replicates. B, Red fluorescent phenotype when viewed under UV light of *fah1-7* mutants compared with Landsberg *erecta* (Ler) control plants grown for 17 days at 50 μmol m^−2^ s^−1^. C, Red fluorescent phenotype under UV light of *fah1-7* mutants grown for 17 days at 50 μmol m^−2^ s^−1^ (low light grown, LL), or 7 days 50 μmol m^−2^ s^−1^ followed by 10 days at 300 μmol m^−2^ s^−1^ (high light grown, HL). D, Representative images of calcofluor white-stained first true leaf cross-sections of LL- and HL-grown *fah1-7* mutant and control plants. Example palisade cells are outlined in red. E, Palisade mesophyll cell height to width ratio from the first leaf pair of plants grown as in (C) and (D). F, Representative fluorescence image of a leaf cross-section from a 17-day-old T1 plant carrying a *IQD22*_pro_:*FAH1-mCit* transgene (*n* = 6). FAH1-mCit, green; chlorophyll autofluorescence, blue. G and H, Representative image (G) and quantification (H) of the relative change in red fluorescence of T1 generation 17-day-old LL-grown *fah1-7* mutant plants carrying transgenes driving either *FAH1-mCit* or a control cDNA (*GUS-mCit*) under the *IQD22* promoter. *P* = 0.006, paired *t* test. Scale bar (G): 5 mm. In (D) and (F), scale bars: 50 μm; adaxial is up. In (E) and (H), boxes indicate 25th and 75th percentiles, horizontal lines the median. Tukey whiskers are shown.

In our FAC sorting studies, we observed that *FAH1* had increased expression in palisade cells relative to other photosynthetic cell types, similar to other phenylpropanoid pathway genes (Figure 6A). Leaves of *fah1* mutants in our conditions also exhibited the previously reported red fluorescent phenotype under UV light (Figure 6B). We further observed a reduction in red fluorescence when plants were grown under high light, likely due to greater sinapoylmalate production as a result of higher expression of other genes in the pathway under this condition (Figure 6C). This is consistent with our RNA-seq results suggesting higher activity of the phenylpropanoid pathway in high light (Figure 5E and Supplemental Figure S9E).

To test the role of phenylpropanoid biosynthesis in the palisade, we first examined if changes in palisade shape in response to high light are altered in *fah1* mutants. However, we found no defect in the cell height to width ratio under both low and high light conditions (Figure 6, D and E). This suggests that neither sinapoylmalate nor the secondary cell wall component S lignin are required for palisade cell shape changes.

Alternatively, phenylpropanoid biosynthesis in the palisade may be important for sinapoylmalate accumulation. Interestingly, the abaxial surface of Arabidopsis wild-type leaves exhibits greater red fluorescence under UV light, suggesting that sinapoylmalate accumulates to greater levels on the adaxial side (Ruegger et al., 1999). While adaxial accumulation of sinapoylmalate in Arabidopsis has generally been assumed to be within the epidermis (Ruegger et al., 1999), in other species ∼50% of UV-B absorbing compounds in the leaf have been found in mesophyll (Day et al., 1996).

**Figure 7:**
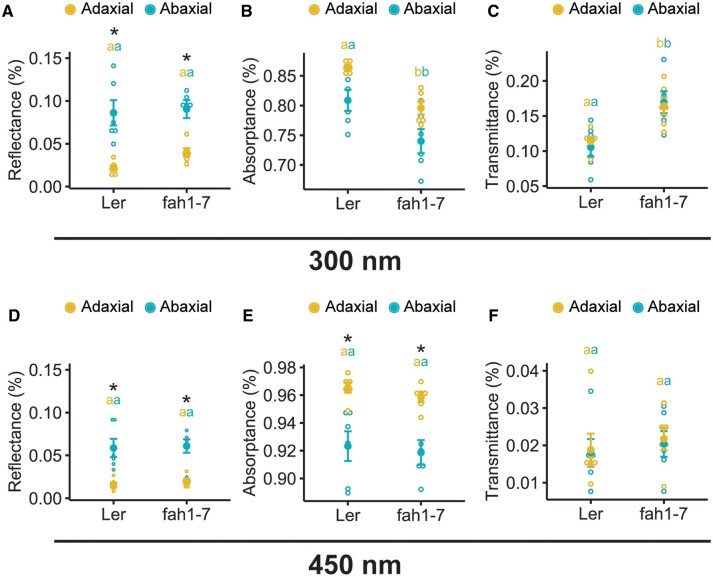
Leaf optical properties are consistent with higher sinapoylmalate production on the adaxial side. Reflectance (A, D), absorptance (B, E), and transmittance (C, F) of first true leaves of 17-day-old Arabidopsis plants under adaxial (yellow) and abaxial (blue) illumination with UV-B light at 300 nm (A–C) or blue light at 450 nm (D–F). *n* = 6 leaves per genotype, indicated by open circles; mean ± sem indicated by filled circles and error bars. Genotypes with different lowercase letters have statistically significant differences (*P* ≤ 0.05, nested ANOVA and Tukey’s HSD test). Asterisks indicate significant (*P* < 0.05) differences in leaf optical properties when illuminated on the adaxial compared with the abaxial leaf surface.

Here, we find that restoring *FAH1* activity specifically to palisade cells of *fah1* mutants by driving expression of the cDNA under the *IQD22* promoter (Figure 6F) rescued the defective red fluorescent phenotype under UV light (Figure 6, G and H). In addition, we found that leaf optical properties are consistent with adaxial sinapoylmalate production. Specifically, compared with wild-type control plants, the leaves of *fah1* mutants have decreased UV light absorptance and increased transmittance (Figure 7, A–C). These defects were specific for UV light; for example, *fah1* mutants displayed minimal differences to wild-type plants in blue wavelengths (Figure 7, D–F). Importantly, less UV light reflectance was also observed on the adaxial surface compared with the abaxial side of both wild-type and mutant plants (Figure 7A), suggesting higher sinapoylmalate levels on the adaxial side. However, a similar observation was seen for blue light (Figure 7D), and, as such, we cannot rule out that other properties of the leaf contribute to this observation. Despite this, our collective results suggest that *FAH1* activity in the palisade is required for adaxial production of sinapoylmalate.

## Discussion

Despite the undisputed importance of palisade mesophyll for optimal leaf performance in many plants, we know surprisingly little about the unique molecular signature of these cells. To this end, we leveraged the power of cell-specific transcriptional profiling using both FAC sorting and scRNA-seq, and identified a previously unappreciated role for the phenylpropanoid pathway in the palisade for production of the UV protectant sinapoylmalate. Our results do not rule out additional production of sinapoylmalate in the adaxial epidermis, where phenylpropanoid gene reporters are also weakly expressed (Figure 1I), and we cannot exclude *FAH1* expression in additional cell types to the palisade in younger leaf tissue in our complementation experiment driven by the *IQD22* promoter (Figure 6, G and H and Supplemental Figure S1F). Interestingly, our observation that at least one gene in the phenylpropanoid pathway, *PAL1*, shifts its expression domain toward the abaxial epidermis under prolonged high intensity light (Figure 5F) suggests that different cell types may have more prominent roles to play in sinapoylmalate and flavonoid pigment production depending on the light environment in which the plants are growing (Hughes and Smith, 2007). Some phenylpropanoid pathway gene expression may be driven by the gradient of light down the leaf. However, stronger *PAL1* and *HCT* reporter activity in the palisade relative to the adaxial epidermis—where incident light is highest—suggests some interaction with cell identity or state (Supplemental Figure S3, F and G).

scRNA-seq is a powerful tool for the characterization of all cell types simultaneously in a given organ. A challenge, however, is to translate clusters of transcriptionally unique cells identified through bioinformatic analyses into verified biological cell identity or transcriptional state. Here, we used biased and unbiased approaches to assign cell types to our scRNA-seq atlas of Arabidopsis leaf protoplasts. Using these methods, we identified upwards of ten different cell types in the mature leaf. In actuality, this number is likely higher, as we did not completely resolve all vascular cell types (see, e.g. Kim et al., 2021) and did not detect or resolve trichome cells. Regardless, this number of cell types is not that dissimilar from some animal organs with their own dedicated functions; for example, the human liver has only around five major cell types, while the kidney contains 20–30 (Quaggin and Phimister, 2016; Trefts et al., 2017). As scRNA-seq technology expands to cover additional vascular plant species, it will be interesting to see just how many leaf cell types identified through transcriptional profiling will be conserved or novel to particular plant clades. For example, in addition to different modes of photosynthesis (Yamori et al., 2013), some plants have evolved novel leaf functions and cell types of which we understand little at the transcriptional level. This includes glandular trichomes for the secretion of biochemicals to protect from herbivores (Huchelmann et al., 2017), hydrenchyma tissue for water storage in succulents (Griffiths and Males, 2017), and mechanosensory leaf structures that sense insect prey in plant carnivores (Lloyd, 1942).

We found similar although not completely overlapping sets of genes with palisade-enriched expression using FAC sorting or scRNA-seq. This may reflect differences in our filtering and analysis methods between the two approaches, differences in the reference photosynthetic cell populations used, the presence of contaminating cells in our FAC-sorted populations, or limitations with scRNA-seq methods, particularly in the identification of low abundance transcripts (Saliba et al., 2014). Of note, we were unable to conclusively identify the palisade cells in our scRNA-seq UMAP plot without taking advantage of the presence of the *IQD22*_pro_:*GUS-mCit* reporter transcript in the background, which provided a molecular handle on the cells. Similarly, other scRNA-seq studies of the Arabidopsis leaf have to date been unable to differentiate the palisade cell population from other mesophyll cells (Liu et al., 2020; Kim et al., 2021; Lopez-Anido et al., 2021). Finally, we also identified a greater number of phenylpropanoid-related genes as being more highly expressed in the palisade relative to other photosynthetic cell types using RNA-seq of bulk FAC sorted cells, perhaps due to the low expression of some of these genes (Supplemental Figure S8). These findings show the limitations of scRNA-seq methods, particularly for cell types that are poorly characterized, share transcriptional similarity to other cell types and/or for which few marker genes exist in the literature. Indeed, caution must be taken when interpreting bioinformatic analyses using scRNA-seq that may include mixed cell populations. In such cases, traditional FAC sorting methods may be more informative, provided a robust and specific fluorescent marker for the cell type of interest is available. Nevertheless, in our hands, both FAC sorting and scRNA-seq methods identified some common genes encoding components of the phenylpropanoid pathway and demonstrate the utility of different approaches to query specific cell types.

One surprising finding was the lack of readily identifiable palisade markers, and instead a diversity of gene expression patterns in the leaf mesophyll. For example, promoter elements from *SQE6*, *PAL1* and *XTH6* all drove expression in the palisade; however, each had additional expression in nonoverlapping sets of other leaf cell types (Figure 1, F–I). This diversity suggests that few genes with strict palisade-specific expression may exist. This observation, coupled with the high degree of similarity we have likely observed between mesophyll types, may have affected our and others’ ability to easily resolve a palisade-specific cluster using scRNA-seq.

Finally, our data describe resources and novel gene promoter elements for those interested in probing palisade cell biology and other leaf cell types. Future work will be necessary to elucidate how photochemistry of the palisade differs, what molecular factors are necessary for regulating and altering palisade cell shape, and ultimately how palisade shape and chemistry interact with other cells to optimize photosynthesis and leaf performance across diverse environments. Our finding here that the palisade layer likely plays a role in protecting the leaf from photooxidative UV damage by increased sinapoylmalate production highlights the utility of unbiased transcriptional profiling experiments like ours to uncover novel aspects of mesophyll biology.

## Materials and methods

### Plant materials and growth conditions

Arabidopsis strains used were Columbia-0 (Col-0), Ler, UCR6 (Geisler et al., 2002), JR11-2 (Gardner et al., 2009), and *fah1-7* (Meyer et al., 1996). For FAC sorting, JR11-2 was introgressed into the Col-0 background eight times. Seeds were sterilized, stratified, germinated, and grown in constant white light (50 μmol m^−2^ s^−1^ unless otherwise indicated; Cool White fluorescent bulbs) on 0.7% agar with 0.5× Linsmaier and Skoog salts and vitamins, on reflective aluminum foil, at 22°C.

### Identification of the UCR6 enhancer trap insertion site

iPCR was used to map the insertion site of the *DsE* enhancer trap transposable element in the UCR6 line using previously described methods (Sundaresan et al., 1995; Geisler et al., 2002). The *DsE* element includes the *GUS* reporter gene fused to a *35S* minimal promoter of CaMV, as well as the *NPTII* gene conferring kanamycin resistance. Briefly, gDNA extracted from UCR6 plants was digested with restriction enzyme (RE) BfaI. The digested product was purified and circularized using T4 DNA ligase, which then served as a template for PCR with primers Ds5O and Ds5I (see Sundaresan et al., 1995; all primer sequences are also shown in Supplemental Data Set S42). This reaction generated a single product of ∼1 kb. Sanger sequencing of the PCR product was used to identify the insertion site of the *GUS* transposable element at position −706 relative to the ATG start site of the *IQD22* gene. This was verified on the other side of the *DsE* element by PCR amplification from UCR6 gDNA template with primers that annealed in the *GUS* and *IQD22* coding sequences (primers CP0190 and CP0194, respectively). On this side, the *GUS*-coding element was inserted at position −715 relative to the *IQD22* start site. To verify that this genomic region was capable of driving palisade-specific expression, we made reporter construct pCP13.11 to replicate the genomic context of the UCR6 *GUS* insertion (see Supplemental Figure S1A). This construct was made using a multistep process:

Regions spanning the *DsE* insertion site were cloned into pBluescript KS(+) (pBS) to create plasmids pCP12.44 and pCP12.45. First, ∼4 kb was PCR amplified using primers CP0241 and CP0244 from Col-0 gDNA template, and ligated into pBS at ApaI/SacI RE sites. These primers inserted into the *IQD22* promoter sequence an additional 3 bp, generating an ApaI site to facilitate the cloning process. Sanger sequencing of the insert revealed a number of differences between the cloned amplicon and the published TAIR10 sequence; however, direct Sanger sequencing of PCR products from our Col-0 gDNA template showed these same base differences in our wild-type Col-0 strain. Next, we inserted into this plasmid a ∼2 kb PCR product amplified from Col-0 gDNA using primers CP0239 and CP0240 at KpnI/ApaI sites. Finally, we inserted one of two PCR fragments amplified from Col-0 gDNA template: (1) a ∼1.5 kb fragment generated using primers CP0245 and CP0246 at a single SacI site to create plasmid pCP12.44 or (2) a ∼1 kb fragment generated using primers CP0237 and CP0238 at the single KpnI site to create plasmid pCP12.45. The orientation of these inserts was confirmed by RE digest and Sanger sequencing.

From this, we generated Gateway vectors (Invitrogen) including enhancer regions 5′ and 3′ of the UCR6 *DsE GUS* insertion site, and which also included either the *35S(−45)* minimal promoter or *Nos* terminator (*NosT*) sequences, respectively. The *35S(−45)* minimal promoter was amplified using primers CP0273 and CP0274 and inserted into pBS at NgoMIV/KpnI RE sites. The 5′ flanking region of the UCR6 *DsE* insertion site was then PCR amplified from pCP12.44 plasmid template using primers CP0296 and CP0298 and inserted at BamHI/SacII RE sites. From this, a 5′ UCR6 enhancer-*35S(−45)* fragment was then amplified using primers CP0299 and CP0300, and recombined with Gateway vector pDONR P4-P1R to create plasmid pCP12.52. Similarly, for the enhancer region 3′ of the GUS insertion site, we first amplified the *NosT* sequence using primers CP0216 and CP0217 from Gateway plasmid pK7m34GW template, and inserted this into pBS at BamHI/SpeI RE sites. The 3′ region of the UCR6 *DsE* insertion site was PCR amplified from pCP12.45 using primers CP0290 and CP0291 and inserted at BamHI/XmaI sites. From this, a *NosT*-3′ UCR6 enhancer fragment was then amplified using primers CP0301 and CP0302 and recombined with Gateway vector pDONR P2R-P3 to create plasmid pCP12.51. The sequences of both pCP12.52 and pCP12.51 were verified by Sanger sequencing.

Finally, to create reporter construct pCP13.11, we recombined pCP12.51, pCP12.52, and pDONR221 carrying *mCit* with Gateway destination vector pK7m34GW.

### Generation of *IQD22*_pro_:*GUS-mCit* reporter plants

Approximately 3.5 kb of *IQD22* promoter sequence was PCR amplified from plasmid pCP12.44 template using primers CP0293 and CP0218, and recombined with Gateway vector pDONR P4-P1R to create plasmid pCP12.46. Clones were verified using Sanger sequencing. To create *IQD22*_pro_:*GUS-mCit* reporter construct pCP13.42 (Supplemental Figure S1A), pCP13.41 was recombined with pDONR221 carrying *GUS* (Procko et al., 2016), pDONR P2R-P3 carrying *mCit* (Jaillais et al., 2011) and the destination vector pK7m34GW. Col-0 plants were dipped with Agrobacterium strain GV3101 carrying pCP13.42, and T2 and T3 seeds tested for single insertion, homozygous segregation patterns. A representative line with strong expression (line #3) was chosen for all subsequent analyses.

### Generation of marker gene transcriptional reporters

Approximately 1.5–2 kb promoter fragments of marker genes identified from FAC sorting and scRNA-seq were PCR amplified using high-fidelity Phusion enzyme (New England BioLabs, Ipswich, Massachusetts, USA) and recombined with Gateway plasmid pDONR P4-P1R. See Supplemental Data Set S42 for primer sequences. Amplification of the correct promoter elements was verified by RE digests and Sanger sequencing with M13F(-21) and M13R primers. Due to the number of sequences being cloned, the promoter fragments were not completely sequenced, and PCR-induced errors in some constructs or variation from the TAIR10 sequence due to differences in our Col-0 strain (e.g. see above) may exist. The plasmids carrying the promoter elements were then recombined with pDONR 221 carrying *GUS*, pDONR P2R-P3 carrying *mCit*, and the destination vector pK7m34GW. Col-0 plants were dipped with Agrobacteria carrying the various reporter constructs. Kanamycin-resistant T1 plants growing on medium containing 50 μg/mL kanamycin were imaged.

### Cloning of additional constructs

To create the *UAS*:*GUS* reporter shown in Supplemental Figure S2F, a pDONR P4-P1R plasmid carrying 5x *UAS* sequences was recombined with pDONR221 carrying *GUS* (Procko et al., 2016), a pDONR-P2RP3 plasmid carrying a coding sequence for a 6xHis-3xFlag tag, and destination vector pB7m34GW. To generate the *IQD22*_pro_:*FAH1-mCit* construct shown in Figure 6, the *FAH1* coding sequence was amplified from Col-0 cDNA template using primers CP1550 and CP1551, and recombined with pDONR221. This was then recombined with pCP12.46, pDONR P2R-P3 carrying *mCit*, and destination vector pK7m34GW to generate plasmid pCP20.124.

### Leaf protoplasting, FAC sorting, and RNA extraction

Protoplasts from the first pair of true leaves were generated using previously described methods (Bargmann and Birnbaum, 2010). Leaves were cut into pieces and placed into protoplasting solution (1.25% w/v Cellulase [Yakult, Tokyo, Japan], 0.3% w/v Macerozyme [Yakult, Tokyo, Japan], 0.4 M D-mannitol, 20 mM MES, 20 mM KCl, 10 mM CaCl_2_, pH 5.7) with gentle agitation for 2 h in a light chamber at 22°C. Each replicate consisted of protoplasts generated from ∼60 leaves. For most experiments, transcription inhibitors actinomycin D and cordycepin were also added to the protoplasting solution at 33 and 100 mg/L, respectively (Leonhardt et al., 2004). The material was then run through a 40 μm cell strainer and the protoplasts pelleted at 500 g for 10 min at 4°C. The protoplast pellet was resuspended in 1 mL ice-cold protoplasting solution without Cellulase and Macerozyme. Protoplasts were immediately sorted. For scRNA-seq, the same method was used, except that RNase inhibitors were added to the protoplasting solution (RNaseOut and SUPERase-In, each at 1:1,000 [Invitrogen]). The protoplast pellet after straining was then washed twice with 0.4 M d-mannitol, 20 mM MES, 20 mM KCl, 33 mg/L actinomycin D, 100 mg/L cordycepin, 1:1,000 RNaseOut, 1:1,000 SUPERase-In, 0.1% BSA, pH 5.7. The first wash also contained 10 mM CaCl_2_. Following the final wash step, the protoplasts were resuspended and collected on a 10 µm strainer (CellTrics 10 µm, sterile [Sysmex]) to allow small debris to flow through. Protoplasts were subsequently washed from the strainer using protoplast buffer and used as input for Single Cell 3′ Gene Expression v3 (10× Genomics) according to the manufacturer’s protocol with the exception that greater than 16,000 protoplasts were used as input. The scRNA-seq library was sequenced on a NovaSeq 6000 (Illumina).

For FAC sorting, mCit+ and GFP+ protoplasts were sorted using the following gating strategy: cells were gated first using forward and side scatter pulse area parameters (FSC-A and SSC-A), aggregates were then excluded using pulse width (FSC-W and SSC-W) before finally gating populations of interest based on red autofluorescence and mCit/GFP fluorescence compared with nontransgenic Col-0 leaf and root protoplast controls. A BD Influx sorter was used, with 1× PBS for sheath fluid, a 100-μm nozzle, and sheath pressure 16.5 PSI. The 1.5-drop Purity sort mode was used. A total of 100,000 protoplasts were sorted into 1.5 mL tubes containing 500 μL Trizol-LS (Thermo Fisher Scientific, Waltham, Massachusetts, USA). Multiple tubes were collected for each sample, with the total number of protoplasts per sample ranging from 200 to 1,100 K. RNA was then extracted using previously described methods (Mertens et al., 2015). Briefly, H_2_O was added to each tube to increase the volume to 750 μL. To this, 250 μL Trizol-LS was added and the tubes gently shaken for 5 min before adding 200 μL chloroform. Tubes were shaken for 5 s, stood for 5 min, and then spun at 12 K rpm using an Eppendorf 5417R refrigerated centrifuge with standard 30-tube capacity fixed-angle rotor for 15 min at 4°C. The aqueous phase was moved to a fresh tube, and 1 μL GlycoBlue (Invitrogen, Waltham, Massachusetts, USA) and 1 mL isopropanol added, mixed, and allowed to stand for 10 min. The RNA was then pelleted at 12 K rpm for 10 min at 4°C. Multiple tubes of the same sample type were pooled at this stage. The RNA pellet was washed with ethanol before air drying and resuspending in 22 μL H_2_O. To this, TURBO DNase was added and inactivated per the manufacturer’s instructions (Invitrogen). Stranded mRNA-seq libraries were prepared using an Illumina TruSeq stranded mRNA library preparation kit according to the manufacturer’s instructions. Libraries were then quantified, pooled, and sequenced at single-end 50-bp reads using the Illumina HiSeq 2500 platform at the Salk Next-Generation Sequencing Core. Raw sequencing data were demultiplexed and converted into FASTQ files using CASAVA (version 1.8.2). Libraries were sequenced at an average depth of 23.7 million reads. All FAC-sorted samples were collected in triplicate.

### Imaging

GUS staining was performed using standard protocols and imaged on a Leica MZ FLIII stereo microscope. Fluorescence images were taken with a Zeiss LSM 710 confocal microscope (mCit: 514 nm excitation, 520–540 nm emission; chlorophyll: 633 nm excitation, 650–720 nm emission; Calcofluor white: 405 nm excitation, 410–520 nm emission). For leaf cross-sections for fluorescence imaging, fresh leaf tissue was embedded in 2% low-melting-temperature agarose for ∼1 h in the dark, and cuts were made along the meso-lateral axis at approximately the middle of the leaf blade. For Calcofluor white staining, cross-sections were placed into 0.1% w/v staining solution, washed with water and imaged. To determine palisade shape, cell height and width were measured using ImageJ from leaf cross section images stained with Calcofluor white or imaged for chlorophyll autofluorescence. In Figure 6E, 12 leaves of each genotype in each condition were sectioned, and 5 palisade cells from each section scored (*n* = 60 cells total for each treatment).

### Leaf red fluorescence under UV light

Seedlings were irradiated with a UVL-56 Blak-ray lamp (Ultra-violet Products, Inc., Cambridge, UK). Images were processed in Photoshop (Adobe). For quantification in Figure 6H, kanamycin-resistant T1 plants growing on 50 μg/mL kanamycin were scored. Because the red fluorescence quickly faded under UV light, an experimental plant was always photographed alongside a control and the red intensity within a 1 mm^2^ region of a first true leaf from each plant recorded using Photoshop and the ratio determined. Plants where the first true leaves were too small to fit a 1 mm^2^ square were not analyzed.

### Leaf optical property measurements

First true leaves were illuminated adaxially and abaxially by a 50W xenon arc lamp (Photon Technology International, Birmingham, New Jersey, USA) emitting wavelengths 200–900 nm. Transmittance (T) was measured using an integrating sphere (4 cm diameter, 3 mm diameter ports) as described by (Gorton et al., 2010) for direct light. Reflectance (R) was measured using a QR600-7-UV-125F reflection/backscatter probe (Ocean Insight) directed at the leaf surface. Spectral information was captured using a USB4000 spectrometer and OceanView software (Ocean Insight). Absorptance (A) was calculated using the relationship 1 = T + R + A. Data were collected at 300 and 450 nm to compare points in the UV and visible spectrum. A nested analysis of variances (ANOVA) was used to test the effect of genotype (main factor) and adaxial or abaxial illumination (nested factor) on leaf T, R, and A. A Tukey’s honest significant difference test (Tukey HSD) was used to examine statistical significance (*P* ≤ 0.05) for pairwise differences between factors. Test results are provided in Supplemental Data Sets S43 and S44.

### Analysis of RNA-seq data from FAC sorted protoplasts

RNA-seq analysis was performed using CyVerse resources (Goff et al., 2011). Raw reads were aligned to the Arabidopsis TAIR10 genome using TopHat version 2.0.9 (strand-specific) (Kim et al., 2013). HTseq version 0.6.1 (mode intersection-strict) (Anders et al., 2014) was used for determining read counts over each gene, and edgeR for differential gene expression (Robinson et al., 2009). In each pairwise comparison, lowly expressed genes with counts < 2 in four or more of the six samples were discarded from the analysis, as were nonnuclear encoded genes. Palisade and nonpalisade reference cells sorted from the same total leaf protoplast pool were paired for statistical analyses where possible. Genes with fold change (FC) > 2 and FDR < 0.05 were classed as differentially regulated.

### scRNA-seq analysis

FASTQ files were generated from BCL files and sequencing reads were aligned to the Araport 11 transcriptome to generate the cell by gene matrix using Cell Ranger version 3.1.0 (10× Genomics, Pleasanton, California, USA). Prior to doublet identification with DoubletDecon (DePasquale et al., 2019), the data were initially filtered based on the number of genes detected and organellar RNA levels to remove both low-quality cells and cells with abnormally high numbers of detected genes, a potential indicator of doublet cells (specifically, cells were removed where detected genes < 650, detected genes > 7,500, mitochondrial reads > 5%, and/or chloroplast reads > 20%). This initial filtering step removed 2,287 cells. The remaining 26,270 cells were then used as input for doublet identification using DoubletDecon, which identified 2,696 putative doublets. In total, 4,983 cells were filtered prior to downstream analysis (17.3% of total cells). Cells containing the highest UMI counts were generally included in the DoubletDecon filtered population. Single cells were then normalized for sequencing depth and UMI coverage of individual cells using sctransform (Hafemeister and Satija, 2019), reads aligned to the chloroplast and mitochondrial genomes were regressed, and single cells were embedded into 2D space by UMAP. Cluster markers were identified using the FindAllMarkers function, comparing expression profiles of cells of individual clusters to all other cells, with cluster markers restricted to transcripts expressed in >25% cells of a cluster at a level of log2 FC > 0.25 and adjusted *P*-value < 0.05. Subclustering analysis was performed identically after subsetting the data for cells of annotated epidermal clusters.

GO term enrichment of filtered cluster markers and gene lists from FAC sorting experiments were performed with clusterProfiler (Wu et al., 2021). Significant GO terms were filtered by *P*-value < 0.05, and functionally redundant GO terms collapsed.

## Accession numbers

RNA-seq raw and processed data have been deposited into the Gene Expression Omnibus with accession numbers GSE182414 and GSE184511.

## Supplemental data

The following materials are available in the online version of this article.

**Supplemental Figure S1.** Identification of a palisade mesophyll-specific regulatory element.

**Supplemental Figure S2.** FAC sorting of palisade and nonpalisade reference cells.

**Supplemental Figure S3.** The complete set of transcriptional reporters (gene promoter:*GUS-mCit*) we generated for a selection of genes identified to be upregulated in the palisade tissue by FAC sorting approaches.

**Supplemental Figure S4.** The complete set of transcriptional reporters (gene promoter:*GUS-mCit*) we generated for a selection of genes identified to be upregulated in the nonpalisade photosynthetic cell population when compared with palisade cells using FAC sorting approaches.

**Supplemental Figure S5.** More stringent filtering does not overly affect our scRNA-seq UMAP-based clustering.

**Supplemental Figure S6.** Expression patterns of de novo-identified scRNA-seq cluster-specific marker genes.

**Supplemental Figure S7.** Subclustering of our leaf scRNA-seq data uncovers additional cell types or cell transcriptional states.

**Supplemental Figure S8.** scRNA-seq fails to detect some lowly expressed transcripts enriched in the palisade.

**Supplemental Figure S9.** Effect of high light on *IQD22* reporter and palisade-associated gene expression.

**Supplemental Table S1.** Comparison of recent scRNA-seq data sets from plants.

**Supplemental Data Sets S1 and S2.** Genes upregulated and downregulated in the palisade relative to other photosynthetic cell types.

**Supplemental Data Sets S3–S19.** scRNA-seq UMAP cluster 0–19 markers.

**Supplemental Data Sets S20–S36.** GO enrichment analysis for clusters 0–16.

**Supplemental Data Set S37.** Genes upregulated in IQD22pro:GUS-mCit transgene positive cells (TransG+) relative to CORI3 positive (CORI3+) cells using scRNA-seq.

**Supplemental Data Set S38.** Genes upregulated in the palisade by increased light intensity.

**Supplemental Data Set S39.** Genes upregulated in nonpalisade photosynthetic cells by increased light intensity.

**Supplemental Data Set S40.** Genes downregulated in the palisade by increased light intensity.

**Supplemental Data Set S41.** Genes downregulated in nonpalisade photosynthetic cells by increased light intensity.

**Supplemental Data Set S42.** Primers used in this study.

**Supplemental Data Set S43.** ANOVA test results for Figure 7.

**Supplemental Data Set S44.** Tukey HSD test results for Figure 7.

## Supplementary Material

koac167_Supplementary_Data

## Notes

All authors contributed to experimental design. T.L. performed the scRNA-seq analysis. A.B. performed the leaf optical property measurements. Other experiments performed by C.P. with assistance from B.O.R.B. and C.O. on FAC sorting, L.B. for leaf staining, J.R.N. with sequencing, and T.D. with molecular cloning. C.P., T.L., and A.B. wrote the manuscript. All other authors provided comments.

The authors responsible for distribution of materials integral to the findings presented in this article in accordance with the policy described in the Instructions for Authors (https://academic.oup.com/plcell) are: Carl Procko (procko@salk.edu) and Joanne Chory (chory@salk.edu).
