# Supplementary material for: Leaf cell-specific and single-cell transcriptional profiling reveals a role for the palisade layer in UV light protection
Source: Plant Cell. 2022 Jun 6;34(9):3261–79. doi: 10.1093/plcell/koac167 (PMC9421592; doi:10.1093/plcell/koac167)
Supplement: koac167_Supplementary_Data [file koac167_supplementary_data.zip › Procko_Supplemental.pdf]

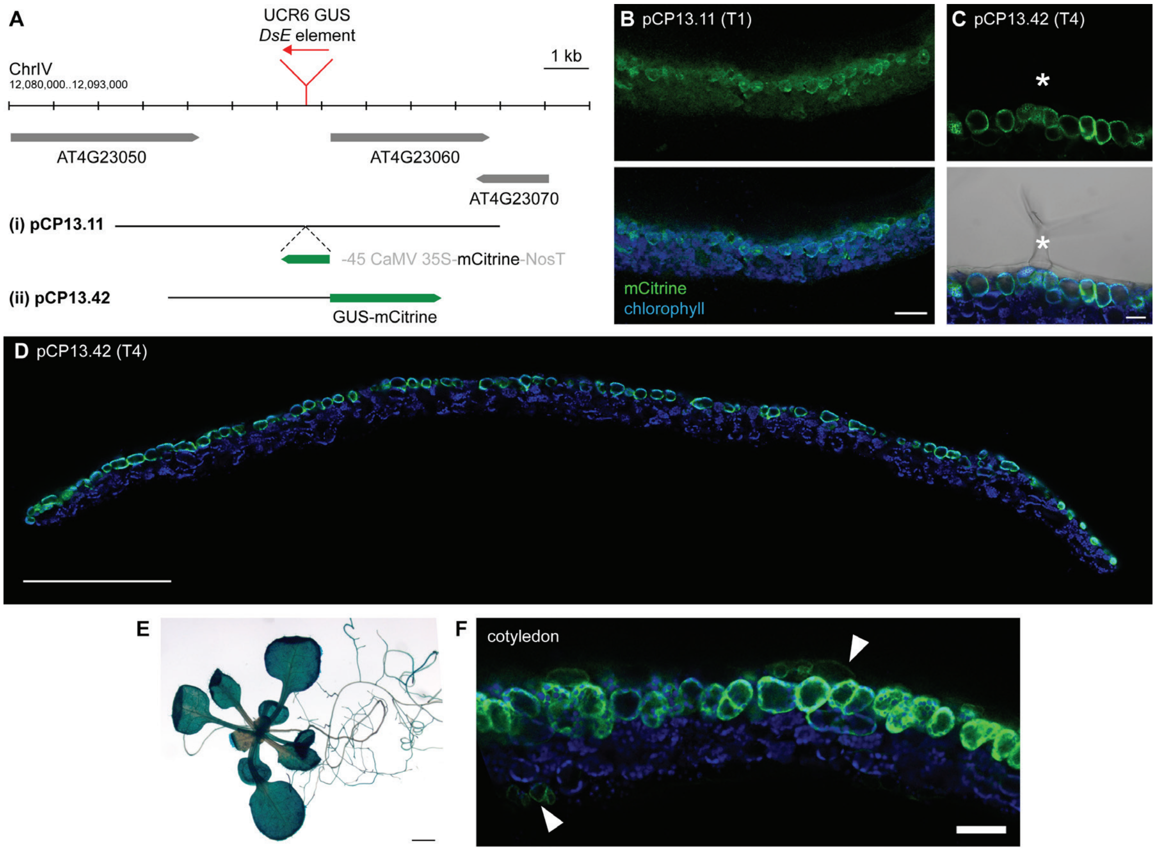


**Supplemental Figure S1.** Identification of a palisade mesophyll-specific regulatory element. A, Mapped insertion site of the *GUS DsE* transposable element in the enhancer trap line UCR6 (Geisler et al., 2002). Black line shows a region on chromosome IV, while red lines indicate the insertion site. The red arrow shows the direction of the *GUS* coding sequence. Gene models in the region are indicated by gray block arrows. *AT4G23060* is the *IQD22* gene. Below, schematic of transcriptional reporters tested in transgenic plants. Construct pCP13.11 (i) includes ~4 kb of 5' and 3' DNA sequences flanking the UCR6 *GUS* insertion site, with a -45 CaMV *35S* minimal promoter:*mCit*:*NosT* terminator sequence (green block arrow) inserted between the flanking regions in the same orientation as the UCR6 *GUS* element. The second construct, pCP13.42 (ii), includes ~3.5 kb of promoter sequence upstream of the *IQD22* start site, fused to the coding sequence for a GUS-mCit fusion protein (green block arrow; referred to in the main text as *IQD22*$_{pro}$:*GUS-mCit*). B, Cross-section of first true leaf showing pCP13.11 *GUS-mCit* reporter activity (green, top) and overlaid with chlorophyll autofluorescence (blue, below) in a T1 transgenic Arabidopsis plant. Note expression in the palisade. C, Cross-section of first true leaf showing *GUS-mCit* reporter activity of a T4 Arabidopsis plant carrying the pCP13.42 (*IQD22*$_{pro}$:*GUS-mCit*) reporter 17 d post-germination. Reporter activity, green; chlorophyll autofluorescence, blue; transmitted light, gray. GUS-mCit fluorescence is detected in palisade cells, including palisade-like cells at the base of trichomes (asterisk marks trichome). D, Same as in panel C, except image shows across the entire leaf plane. Line 3 (pictured), was used for FAC sorting experiments. E, GUS stain of 17-d-old plants carrying the pCP13.42 reporter, showing additional expression in roots and petioles. F, Cross-section of cotyledon of 4-d-old plants carrying the pCP13.42 (*IQD22*$_{pro}$:*GUS-mCit*) reporter. Note strong expression in palisade cells and some weak expression in the epidermis (arrowheads) for this tissue and at this age. Scale bars: B, 100 µm; C and F, 50 µm; D, 500 µm; E, 2 mm. In cross-sections, adaxial is up. Supports Figure 1.

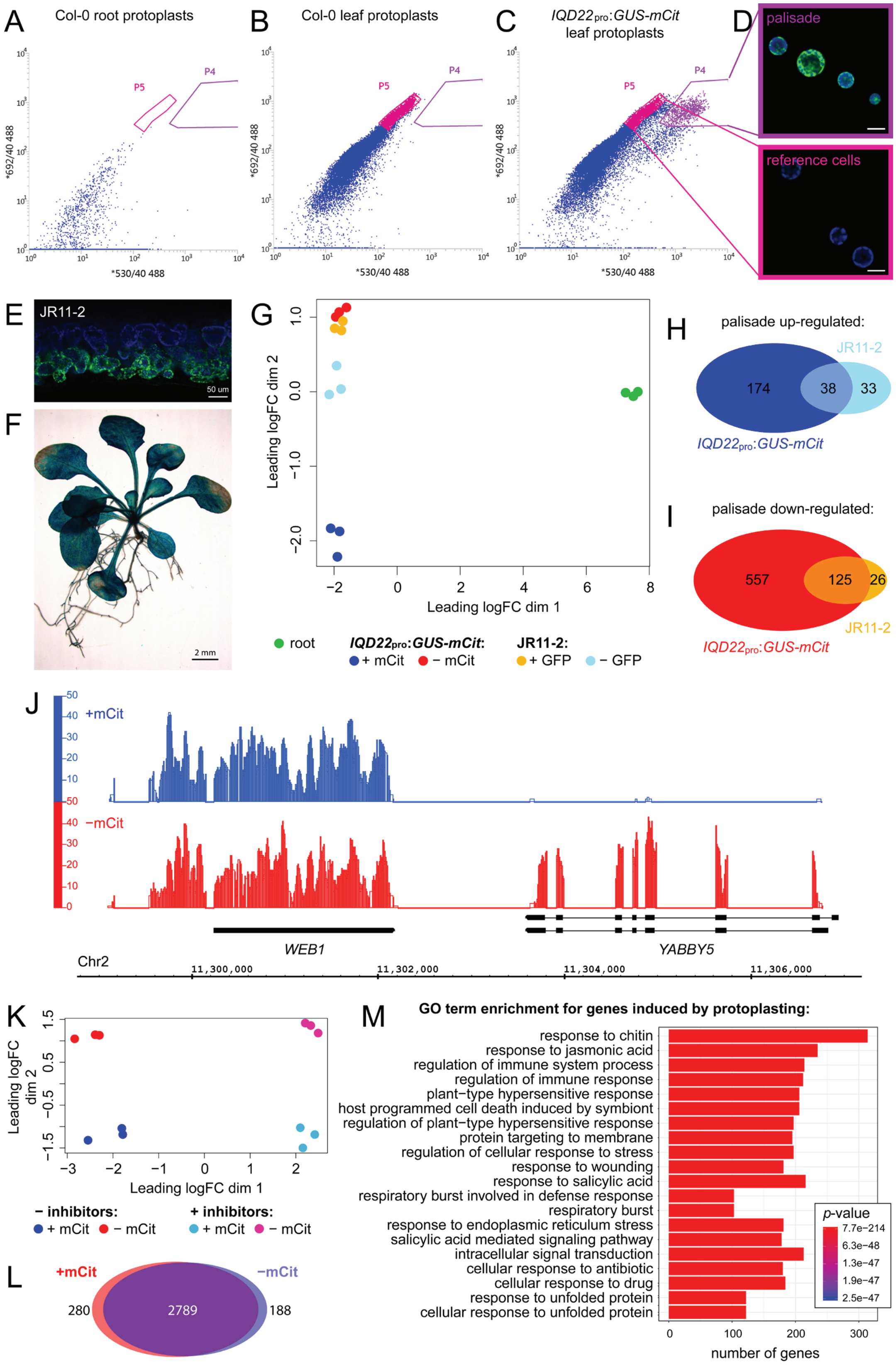


**Supplemental Figure S2.**

**Supplemental Figure S2.** FAC sorting of palisade and non-palisade reference cells. A-C, Representative dot plots of root (A) and leaf (B) protoplasts from wild-type Col-0 plants, and leaf protoplasts of *IQD22*$_{pro}$:*GUS-mCit* transgenic plants (C) showing green/yellow (x-axis) and red (y-axis) fluorescence. Note that protoplasts generated from leaf tissue have more cells with high red fluorescence due to the presence of chlorophyll. In *IQD22*$_{pro}$:*GUS-mCit* plants, a subset of these cells also exhibited high green fluorescence (the palisade population). *n*(events) = 50, 000 (A), 128,803 (B) and 109,305 (C). D, Fluorescence microscopic images of sorted cell protoplast populations used in this study: palisade cells (top, gate P4), defined as having both high red and green fluorescence, and non-palisade photosynthetic cells (bottom, gate P5), having high red fluorescence but lacking mCit green/yellow fluorescence. Gate P4 was set high on the y-axis to avoid as much contamination as possible from non-chloroplast containing cells (e.g. see those in panel A), and gate P5 was set similarly high to appropriately match. Note that in microscopy images chlorophyll autofluorescence is false-colored blue. Scale bars, 20 µm. E, Fluorescence image of a 15 d-old first leaf cross-section from an Arabidopsis plant carrying the JR11-2 enhancer trap, introgressed into the Col-0 background. Chlorophyll autofluorescence, blue, and GFP, green. Note expression predominantly in the lower spongy mesophyll. Adaxial is up. See also (Gardner et al., 2009). F, Example of a GUS-stained 20 d-old JR11-2 plant (C24 background) also carrying a 5x*UAS*:GUS transgene (T2 generation) showing reporter activity across the entire leaf blades. G, Principle components analysis comparing the RNA-seq transcriptional profiles of different protoplast populations. Cell populations include: (i) total root protoplasts (green circles), (ii) a palisade mesophyll-enriched population generated from FAC sorted *IQD22*$_{pro}$:*GUS-mCit* leaf protoplasts exhibiting mCit and chlorophyll fluorescence (dark blue circles), and (iii) a reference photosynthetic cell population from *IQD22*$_{pro}$:*GUS-mCit* leaves having chlorophyll fluorescence but lacking mCit (red circles). Also included are FAC sorted protoplasts from JR11-2 enhancer trap plants: (iv) a palisade-enriched population having chlorophyll but lacking GFP expression (light blue circles), and (v) a reference population having both GFP and chlorophyll fluorescence (orange circles). Each population was collected in triplicate. H and I, Venn diagrams showing a comparison of genes identified with greater (H) or less (I) expression in FAC sorted palisade mesophyll cells compared to chlorophyll-containing reference populations for protoplasts generated from either *IQD22*$_{pro}$:*GUS-mCit* or JR11-2 plants (differentially-regulated genes show > 2-fold difference and FDR < 0.05). The two reporter lines generated similar results ($p < 0.005$ for both H and I; hypergeometric test). J, Sum depth chart showing an example of aligned RNA sequencing reads over the *WEAK CHLOROPLAST MOVEMENT UNDER BLUE LIGHT 1* (*WEB1*) gene and abaxial fate-determining gene *YABBY*5 (Eckardt, 2010) for palisade-enriched (+mCit, dark blue) and chloroplast-containing reference (-mCit, red) protoplast populations generated from *IQD22*$_{pro}$:*GUS-mCit* plants. Gene models are shown in black. Sequencing reads from samples CP1701 and CP1702 are shown (see GEO accession GSE182414). Note that both populations express high levels of *WEB1*, but only the non-palisade reference population expresses *YABBY5*. K, Principle components analysis comparing the RNA-seq transcriptional profiles of protoplast populations generated with or without transcriptional inhibitors during the protoplasting process. Samples include FAC sorted palisade (+mCit) and non-palisade reference cell (-mCit) populations from *IQD22*$_{pro}$:*GUS-mCit* transgenic plants. Samples for each cell population and treatment were collected in triplicate. L, Venn diagram showing the overlap in genes up-regulated (fold-change > 2-fold and FDR < 0.05) in the absence of transcriptional inhibitors in the palisade cells (+mCit) and the reference cell population (-mCit). Transcriptional inhibitors affected many of the same genes in the two cell populations ($p < 0.005$, hypergeometric test). M, Gene ontology (GO) term enrichment analysis for the set of overlapping genes in panel L, arranged by adjusted *p*-value. The top 20 terms are shown. Supports Figure 1.

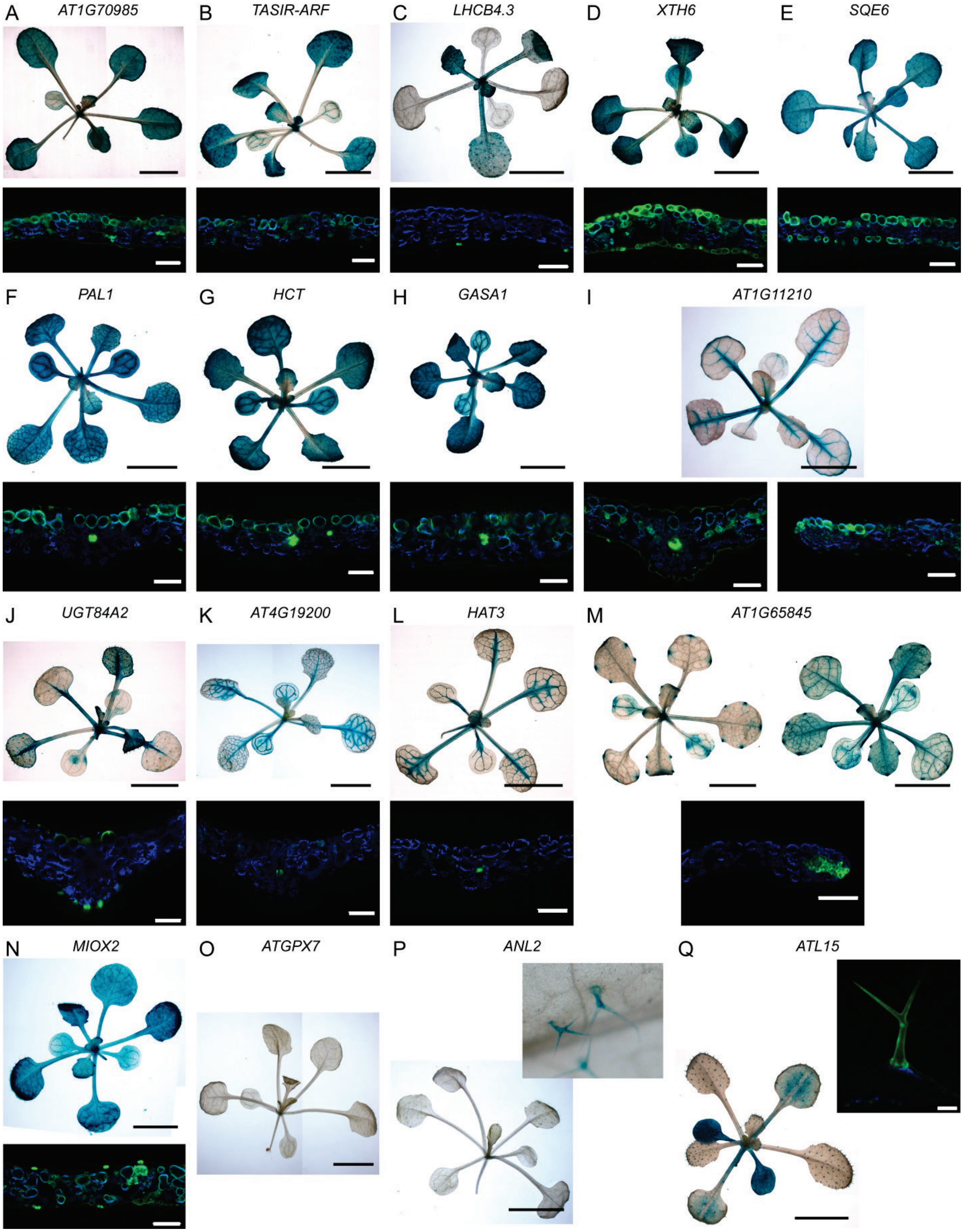


**Supplemental Figure S3.**

**Supplemental Figure S3.** The complete set of transcriptional reporters (gene promoter:*GUS-mCit*) we generated for a selection of genes identified to be up-regulated in the palisade tissue by FAC sorting approaches. Top, GUS stain of whole shoot. Below, fluorescence image of a cross-section through one of the first true leaves (GUS-mCit, green; chlorophyll, blue; adaxial side is up). Plants are 17 d-old. Representative images of T1 plants are shown ($n \geq 4$ for each reporter construct). Promoter elements for *AT1G70985* (A) and *TASIR-ARF* (B) drove weak but specific expression in the palisade. In our hands, the promoter of *LHCB4.3* (C) drove expression in guard cells in the first leaves, although it has been reported by others to express on the adaxial leaf side (Sawchuk et al., 2008). The *XTH6* reporter (D) expressed in epidermal and mesophyll cells, including palisade, with strongest expression on the adaxial leaf side. The *SQE6* promoter (E) drove strong expression in the palisade and lowermost layer of spongy mesophyll. *PAL1* (F), *HCT* (G) and *GASA1* (H) reporters expressed most strongly in vascular cells, with weaker expression localized to the palisade and epidermis. An *AT1G11210* reporter (I) also had strongest expression in the vasculature, with additional weak expression in the palisade over the midvein (left) and at the leaf margins (right). For all other reporters tested, we did not observe localized palisade expression. This may be due to missing *cis* regulatory elements in our reporters, weak expression that was difficult to detect by our imaging methods, or contaminating cell types in our FAC sorted population (see main text). These included reporters for *UGT84A2* (J), which appeared brightest in the epidermal cells closest to the midvein; *AT4G19200* (K) and *HAT3* (L), which expressed most strongly in the veins; *AT1G65845* (M) in the hydathodes, with additional expression localized to veins in some plants (two examples of GUS stained T1 plants are shown); *MIOX2* (N) in guard cells and mesophyll; *ATGPX7* (O), for which no expression was detected; *ANL2* (P) in trichomes; and *ATL15* (Q) in trichomes, with weak GUS staining in other leaf cell types that we were unable to visualize using fluorescence microscopy. Fluorescence images in panels F, G, H, I (bottom left), J, K and L are taken at the midvein. In M, the cross section is at the hydathode. Insets in panels P and Q show close ups of the trichomes. Scale bars: GUS stains, 5 mm; fluorescence images, 100 µm. The fluorescence images shown in panels A, D, E and F are also shown in Figure 1. Supports Figure 1.

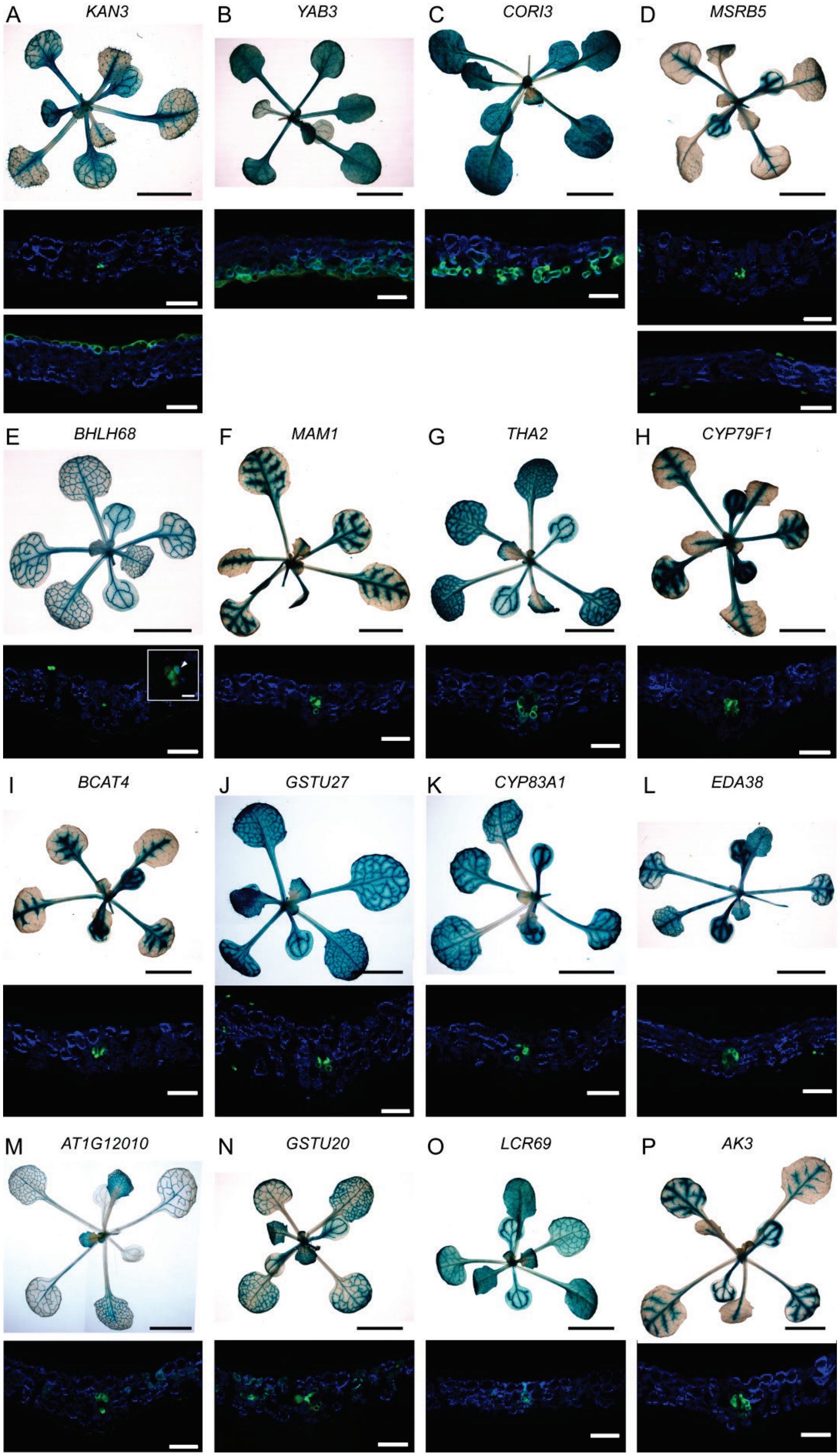


**Supplemental Figure S4.**

**Supplemental Figure S4.** The complete set of transcriptional reporters (gene promoter:*GUS-mCit*) we generated for a selection of genes identified to be up-regulated in the non-palisade photosynthetic cell population when compared to palisade cells using FAC sorting approaches. Top, GUS stain of whole shoot. Below, fluorescence image of a cross-section through one of the first true leaves (GUS-mCit, green; chlorophyll, blue; adaxial is up). Plants are 17 d-old. Representative images of T1 plants are shown ($n \geq 4$ for each reporter construct). Surprisingly, a promoter element for the abaxial-specification factor *KAN3* (A) (Eshed et al., 2004) drove expression in the proximal adaxial epidermis over the midvein in some T1 plants, as well as in veins at this point in developmental time. By contrast, a reporter for another abaxial-specification factor, *YAB3* (B) (Eckardt, 2010), was expressed in abaxial cell types, including the lower epidermis and spongy mesophyll. The promoter for *CORI3* (C) drove reporter expression primarily in the lower spongy mesophyll and chloroplast-containing cells around the vasculature. *MSRB5* (D) and *BHLH68* (E) reporters were expressed in veins and guard cells. Inset in panel E shows presence of chloroplasts (arrowhead) in the GUS-mCit expressing cells in the vein. All other reporters tested drove expression in bundle sheath cells, and possibly other cells of the vein. This included gene promoters *MAM1* (F), *THA2* (G), *CYP79F1* (H), *BCAT4* (I), *GSTU27* (J), *CYP83A1* (K), *EDA38* (L), *AT1G12010* (M), *GSTU20* (N), *LCR69* (O), and *AK3* (P). In addition, *GSTU27* and *EDA38* reporters were expressed in guard cells. All fluorescence images are taken at the midvein except lower panel D. Scale bars: GUS stains, 5 mm; fluorescence images, 100 µm, except inset panel E, 10 µm. The fluorescence images shown in panels C and G are also shown in Figure 1. Supports Figure 1.

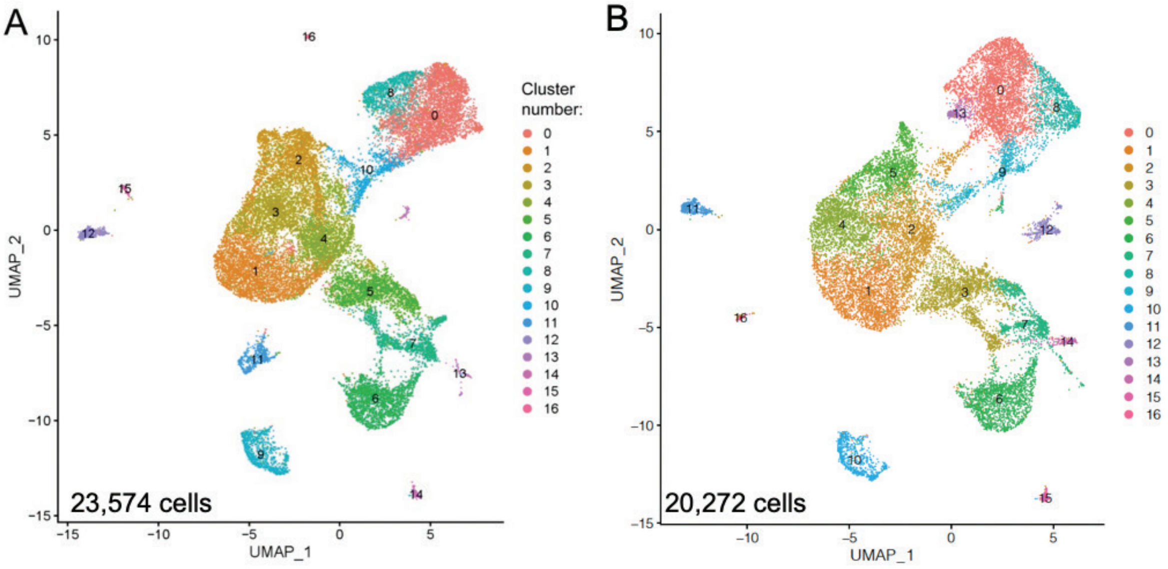


**Supplemental Figure S5.** More stringent filtering does not overly affect our scRNA-seq UMAP-based clustering. A, Unbiased clustering of RNA-sequenced protoplasts following filtering (see Materials and Methods) from first true leaves of 17-d-old *IQD22*pro:*GUS-mCit* plants. See also Figure 2. B, Unbiased clustering of protoplasts following additional filtering of cells with > 10,000 UMI per cell. Note the similarity in the structure of the data, and the similar number of cell clusters identified. Supports Figure 2.

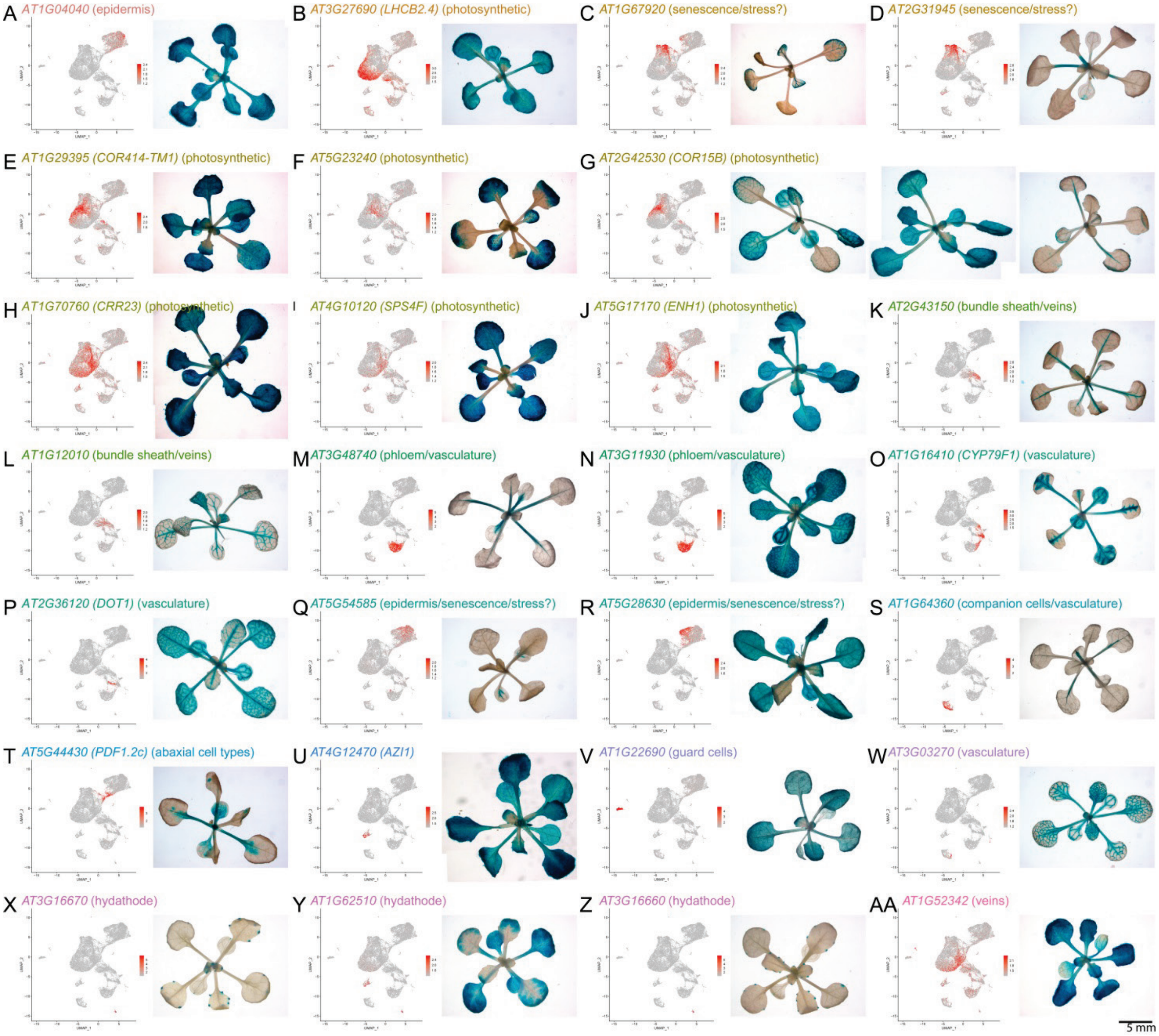


**Supplemental Figure S6.** Expression patterns of *de novo*-identified scRNA-seq cluster-specific marker genes. Left, expression levels of the marker genes mapped to our scRNA-seq data of leaf protoplasts, and right, representative shoot images of 17-d-old T1 GUS-stained transcriptional reporter plants ($n \geq 4$ for each reporter construct). Marker genes are as shown in Figure 3B and C. These include: A, *AT1G04040*; B, *AT3G27690* (*LHCB2.4*); C, *AT1G67920*; D, *AT2G31945*; E, *AT1G29395* (*COR414-TM1*); F, *AT5G23240*; G, *AT2G42530* (*COR15B*); H, *AT1G70760* (*CRR23*); I, *AT4G10120* (*SPS4F*); J, *AT5G17170* (*ENH1*); K, *AT2G43150*; L, *AT1G12010*; M, *AT3G48740*; N, *AT3G11930*; O, *AT1G16410* (*CYP79F1*); P, *AT2G36120* (*DOT1*); Q, *AT5G54585*; R, *AT5G28630*; S, *AT1G64360*; T, *AT5G44430* (*PDF1.2c*); U, *AT4G12470* (*AZI1*); V, *AT1G22690*; W, *AT3G03270*; X, *AT3G16670*; Y, *AT1G62510*; Z, *AT3G16660*; and AA, *AT1G52342*. For panel G, *AT2G42530* (*COR15B*), variation existed between the T1 reporter plants imaged, and three examples are shown with different expression patterns and/or levels. Gene name colors are the same as the color of the cell cluster in Figure 2A for which the marker gene was identified. The likely cell identity for each cluster is indicated in parentheses. All microscopy images are at the same scale. Reporters for *AT1G12010* and *AT1G16410* (*CYP79F1*) are also shown in Supplemental Figure S4. Supports Figures 2 and 3.

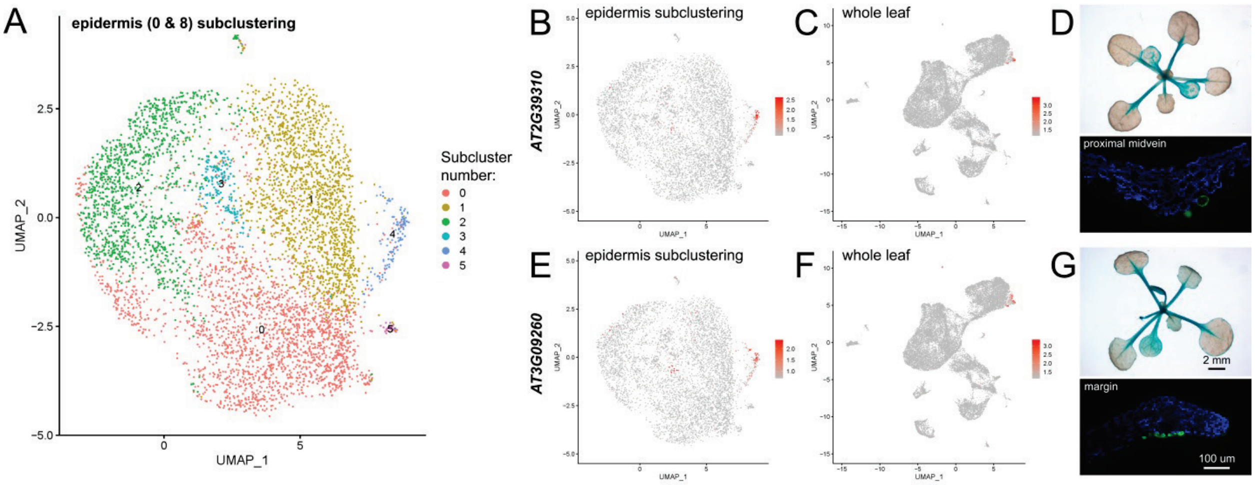


**Supplemental Figure S7.** Subclustering of our leaf scRNA-seq data uncovers additional cell types or cell transcriptional states. A, Subclustering of protoplasts assigned to epidermal cell clusters 0 and 8 (see Figure 2A) identified six subclusters. B-G, Analysis of two subcluster marker genes, *AT2G39310* and *AT3G09260*, with enriched expression within subcluster 4. Expression levels were mapped to the subclustered data (B and E) and entire dataset (C and F). Representative images of GUS-stained shoots (top) and fluorescence images of first true leaf cross-sections at the midvein and margin (bottom) of 17-d-old T1 transcriptional reporter plants are also shown (D and G). In fluorescence images, GUS-mCit is green, chlorophyll autofluorescence blue, and adaxial is up. Note mCit fluorescence in the lower epidermal pavement cells at the midvein and leaf margin. Scales in D and G are the same. Supports Figure 2.

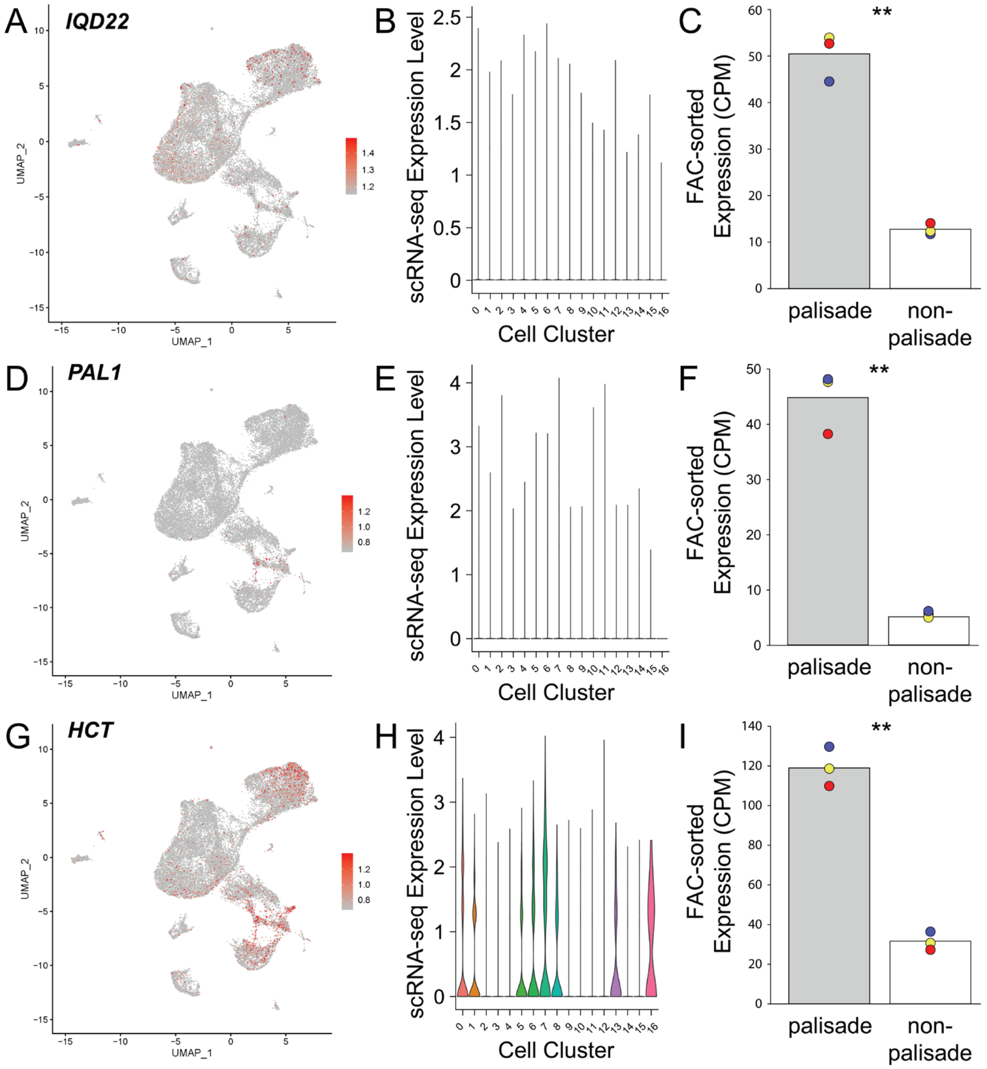


**Supplemental Figure S8.** scRNA-seq fails to detect some lowly-expressed transcripts enriched in the palisade. Expression maps (A, D, G) and violin plots (B, E, H) showing gene expression levels in our scRNA-seq UMAP-based plot and associated cell clusters, compared to expression levels in the palisade cell population versus non-palisade reference cells generated from FAC sorting (C, F, I), for our palisade-marker gene *IQD22* and phenylpropanoid pathway-associated genes *PAL1* and *HCT*, respectively. Note that *IQD22* and *PAL1* were poorly detected by scRNA-seq methods in mesophyll-associated clusters 1-4, even though transcriptional reporters for all three genes had robust palisade expression (see Figure 1C and I; Supplemental Figure S3G) and were detected in the palisade by FAC sorting. In (C, F, I), counts per million (CPM) are shown, and colored points indicate paired replicates. **FDR < 0.005. Supports Figure 4.

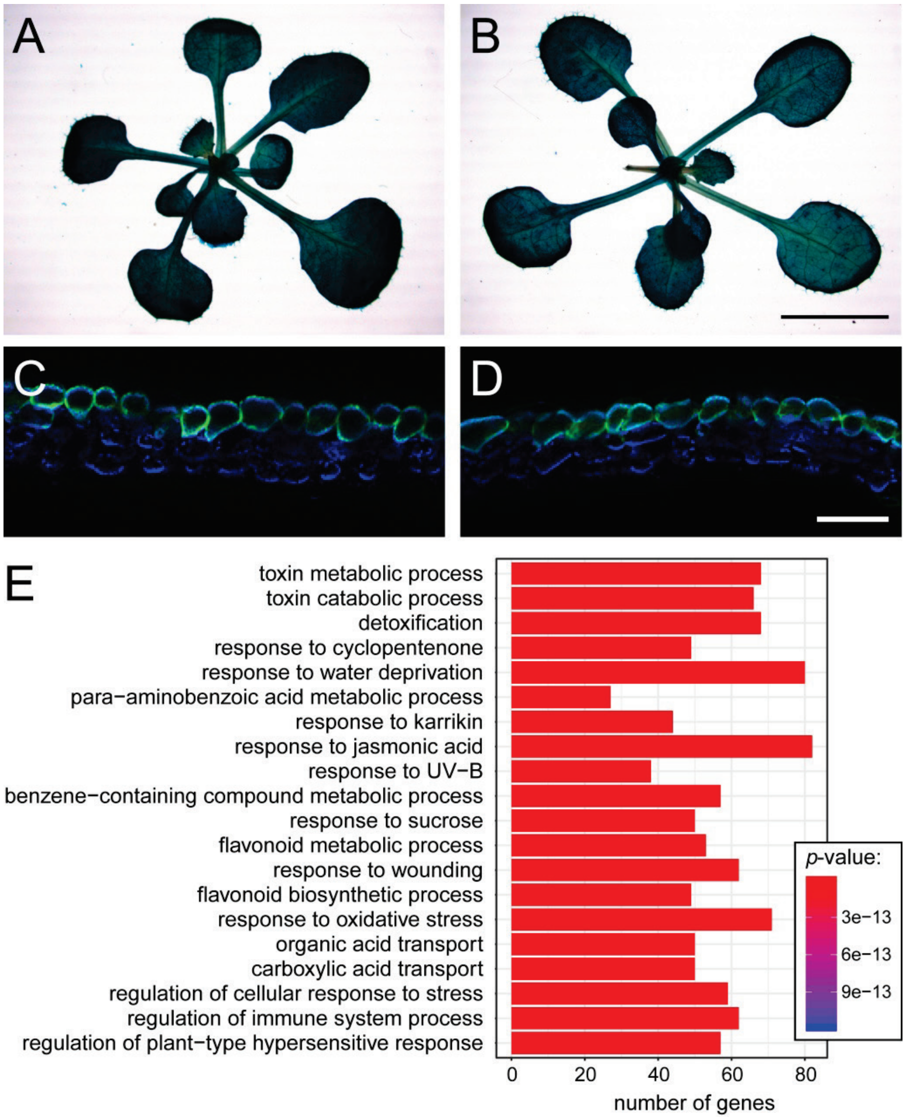


**Supplemental Figure S9.** Effect of high light on *IQD22* reporter and palisade-associated gene expression. A and B, GUS staining in the shoots of 17-d-old $IQD22_{pro}$:*GUS-mCit* reporter plants grown at low light (50 µE $m^{-2}$ $s^{-1}$, A) or treated for 2 h with high light (300 µE $m^{-2}$ $s^{-1}$) immediately prior to protoplasting (B). Scale bar: 5 mm. C and D, Same as in A and B, respectively, except showing a representative cross-section through the first true leaf. GUS-mCit fluorescence, green, and chlorophyll autofluorescence, blue. Scale bar: 100 µm. Adaxial is up. E, Gene ontology (GO) term enrichment analysis of genes up-regulated by 2 h high light in FAC sorted palisade cells. The top 20 GO terms by adjusted *p*-value are shown. Supports Figure 5.

**Supplemental Table S1.** Comparison of recent scRNA-seq data sets from plants.

| Publication | median genes/cell | median UMI*/cell | # cells |
|---|---|---|---|
| This publication | 1,637 | 3,639 | 23,729 |
| Graeff et al., 2021 | 3,525 to 4,214 | not provided | 5,767 to 6,742 |
| Jean-Baptiste et al., 2019 | 2,445 | 6,152 | 3,121 |
| Kim et al., 2021 | 3,342 | 27,159 | 5,230 |
| Lopez-Anido et al., 2021** | 1,870 | 5,026 | 5,021 |
| Shulse et al., 2019 | 919 to 1,497 | 1,454 to 2,976 | 12,000 |
| Wendrich et al., 2020 | 6,781 | not provided | 5,145 |
| Zhang et al., 2019 | 1,875 | not provided | 7,695 |
| Zhang et al., 2021 | 3,360 | not provided | 15,034 |

*Unique molecular identifiers (UMI). **Other data sets from this paper were sequenced more shallowly.

## REFERENCES FOR SUPPLEMENTAL MATERIAL


Eckardt, N. A. (2010). YABBY Genes and the Development and Origin of Seed Plant Leaves. *Plant Cell, 22*(7), 2103-2103. doi:10.1105/tpc.110.220710

Eshed, Y., Izhaki, A., Baum, S. F., Floyd, S. K., & Bowman, J. L. (2004). Asymmetric leaf development and blade expansion in Arabidopsisare mediated by KANADI and YABBY activities. *Development, 131*(12), 2997-3006. doi:10.1242/dev.01186

Gardner, M. J., Baker, A. J., Assie, J. M., Poethig, R. S., Haseloff, J. P., & Webb, A. A. (2009). GAL4 GFP enhancer trap lines for analysis of stomatal guard cell development and gene expression. *J Exp Bot, 60*(1), 213-226.

Geisler, M., Jablonska, B., & Springer, P. S. (2002). Enhancer trap expression patterns provide a novel teaching resource. *Plant Physiol, 130*(4), 1747-1753. doi:10.1104/pp.011197

Graeff, M., Rana, S., Wendrich, J. R., Dorier, J., Eekhout, T., Aliaga Fandino, A. C., . . . Hardtke, C. S. (2021). A single-cell morpho-transcriptomic map of brassinosteroid action in the Arabidopsis root. *Mol Plant, 14*(12), 1985-1999. doi:10.1016/j.molp.2021.07.021

Jean-Baptiste, K., McFaline-Figueroa, J. L., Alexandre, C. M., Dorrity, M. W., Saunders, L., Bubb, K. L., . . . Cuperus, J. T. (2019). Dynamics of Gene Expression in Single Root Cells of Arabidopsis thaliana. *Plant Cell, 31*(5), 993-1011. doi:10.1105/tpc.18.00785

Kim, J.-Y., Symeonidi, E., Pang, T. Y., Denyer, T., Weidauer, D., Bezrutczyk, M., . . . Frommer, W. B. (2021). Distinct identities of leaf phloem cells revealed by single cell transcriptomics. *Plant Cell, 33*(3), 511-530. doi:10.1093/plcell/koaa060

Lopez-Anido, C. B., Vatén, A., Smoot, N. K., Sharma, N., Guo, V., Gong, Y., . . . Bergmann, D. C. (2021). Single-cell resolution of lineage trajectories in the Arabidopsis stomatal lineage and developing leaf. *Dev Cell, 56*(7), 1043-1055.e1044. doi:10.1016/j.devcel.2021.03.014

Sawchuk, M. G., Donner, T. J., Head, P., & Scarpella, E. (2008). Unique and Overlapping Expression Patterns among Members of Photosynthesis-Associated Nuclear Gene Families in Arabidopsis. *Plant Physiol, 148*(4), 1908-1924. doi:10.1104/pp.108.126946

Shulse, C. N., Cole, B. J., Ciobanu, D., Lin, J., Yoshinaga, Y., Gouran, M., . . . Dickel, D. E. (2019). High-Throughput Single-Cell Transcriptome Profiling of Plant Cell Types. *Cell Reports, 27*(7), 2241-2247.e2244. doi:10.1016/j.celrep.2019.04.054

Wendrich, J. R., Yang, B., Vandamme, N., Verstaen, K., Smet, W., Van de Velde, C., . . . De Rybel, B. (2020). Vascular transcription factors guide plant epidermal responses to limiting phosphate conditions. *Science, 370*(6518). doi:10.1126/science.aay4970

Zhang, T.-Q., Chen, Y., & Wang, J.-W. (2021). A single-cell analysis of the Arabidopsis vegetative shoot apex. *Dev Cell, 56*(7), 1056-1074.e1058. doi:10.1016/j.devcel.2021.02.021

Zhang, T.-Q., Xu, Z.-G., Shang, G.-D., & Wang, J.-W. (2019). A Single-Cell RNA Sequencing Profiles the Developmental Landscape of Arabidopsis Root. *Mol Plant, 12*(5), 648-660. doi:10.1016/j.molp.2019.04.004
